# Lego-Inspired Glass Capillary Microfluidic Device: A Technique for Bespoke Microencapsulation of Phase Change Materials

**DOI:** 10.1021/acsami.3c00281

**Published:** 2023-03-24

**Authors:** Sumit Parvate, Goran T. Vladisavljević, Nico Leister, Alexandros Spyrou, Guido Bolognesi, Daniele Baiocco, Zhibing Zhang, Sujay Chattopadhyay

**Affiliations:** † Department of Chemical Engineering, Loughborough University, Loughborough LE11 3TU, United Kingdom; ‡ Polymer and Process Engineering, Indian Institute of Technology, Roorkee, Saharanpur 247001, India; § Institute of Process Engineering in Life Sciences, Karlsruhe Institute of Technology, 76131 Karlsruhe, Germany; ∥ School of Chemical Engineering, University of Birmingham, Birmingham B15 2TT, United Kingdom

**Keywords:** phase change material, hydrated salt, microencapsulation, microfluidics, latent heat storage, photocatalysis, core−shell microcapsules

## Abstract

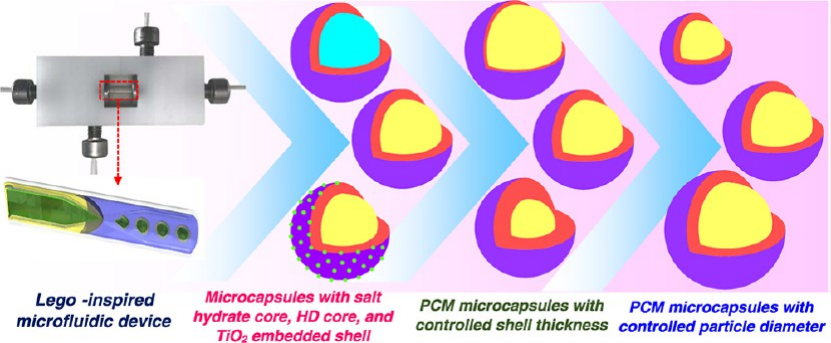

We report a Lego-inspired glass capillary microfluidic device capable of encapsulating both organic and aqueous phase change materials (PCMs) with high reproducibility and 100% PCM yield. Oil-in-oil-in-water (O/O/W) and water-in-oil-in-water (W/O/W) core–shell double emulsion droplets were formed to encapsulate hexadecane (HD, an organic PCM) and salt hydrate SP21EK (an aqueous PCM) in a UV-curable polymeric shell, Norland Optical Adhesive (NOA). The double emulsions were consolidated through on-the-fly polymerization, which followed thiol-ene click chemistry for photoinitiation. The particle diameters and shell thicknesses of the microcapsules were controlled by manipulating the geometry of glass capillaries and fluid flow rates. The microcapsules were monodispersed and exhibited the highest encapsulation efficiencies of 65.4 and 44.3% for HD and SP21EK-based materials, respectively, as determined using differential scanning calorimetry (DSC). The thermogravimetric (TGA) analysis confirmed much higher thermal stability of both encapsulated PCMs compared to pure PCMs. Polarization microscopy revealed that microcapsules could sustain over 100 melting–crystallization cycles without any structural changes. Bifunctional microcapsules with remarkable photocatalytic activity along with thermal energy storage performance were produced after the addition of 1 wt % titanium dioxide (TiO_2_) nanoparticles (NPs) into the polymeric shell. The presence of TiO_2_ NPs in the shell was confirmed by higher opacity and whiteness of these microcapsules and was quantified by energy dispersive X-ray (EDX) spectroscopy. Young’s modulus of HD-based microcapsules estimated using micromanipulation analysis increased from 58.5 to 224 MPa after TiO_2_ incorporation in the shell.

## 1 Introduction

Over the past few years, COVID-19 pandemic and global conflicts have disrupted fossil fuel supplies, which provoked a disparity between energy need and delivery. In this context, the best policy to surmount the energy crisis would be the unification of energy resilience and renewable energy storage technologies. As yet, various thermal energy storage technologies have been developed, including sensible heat storage through a temperature gradient, latent heat storage through phase change materials (PCMs), and chemical energy storage through chemical reactions.^1^ PCMs are attractive due to their high energy storage density and the ability to reversibly absorb and release a tremendous amount of thermal energy at a nearly constant temperature. In particular, solid–liquid PCMs are appealing due to negligible volume change during phase transition, high latent heat, and the optimum range of phase change temperature for the majority of applications. Unfortunately, pure solid–liquid PCMs are prone to leakage when they undergo a solid–liquid phase transition. Furthermore, the majority of pure PCMs can experience additional problems during handling and phase change, such as low thermal conductivity, supercooling, phase separation, evaporation, and corrosion,^2^ which necessitate their modifications. Hence, several techniques for the confinement of PCMs at nanoscale and microscale (core–shell encapsulation, porous confinement, composites, etc.) were suggested to overcome the abovementioned issues with pure PCMs.

Microencapsulation technology offers effective solutions to the inherent problems associated with pure PCMs. It is mainly achieved through suspension polymerization, Pickering emulsion polymerization, interfacial polymerization, in situ polymerization, complex coacervation, ionic gelation, and a sol–gel process.^3^ However, these encapsulation techniques are based on classical emulsification processes that entail top-down emulsifying machines such as sonicators, high-shear rotor–stator machines, or high-pressure homogenizers.^4,5^ In these processes, the PCM emulsion is produced using high energy input and the resulting emulsion, often containing submicron droplets, is then dispersed in the continuous phase. In the second emulsification step, the energy input must be kept rather low to minimize the unintentional release of PCM droplets which is, however, to a certain extent unavoidable. Since top-down emulsification processes involve the breakup of large droplets into smaller ones, these processes are energy-intensive with low encapsulation yields, and the resulting droplets are highly polydispersed.^6^ Thus, microcapsules formed from such emulsions are poorly controlled in size, structure, and functionality. Wide droplet size distribution of both inner and outer droplets prepared using conventional methods can lead to low batch-to-batch reproducibility of the process and that precludes widespread use of these techniques. In addition, conventional encapsulation processes are often multistage and protracted (reaction time ∼ 24 h).^7,8^

Droplet microfluidics can circumvent the anomalies of the bulk emulsification process and generates monodispersed microcapsules with well-defined morphologies. In particular, PDMS chips and disposable glass capillary devices have been extensively explored in PCM microencapsulation.^9^ Capillary microfluidics was introduced in Weitz’s lab at Harvard University,^10,11^ but the first successful microfluidic encapsulation of PCM (*n*-octadecane) in a polyurea shell was performed by Lone et al.^12^ using tubular microfluidics. Soon after, Fu et al.^13^ employed a coflow microfluidic device to produce elastic microcapsules made of silicone as a shell and *n*-hexadecyl bromide (PCM) as a core. Han et al.^14^ reported a capillary microfluidic route for the synthesis of poly(ethylene glycol) diacrylate (PEGDA) microcapsules containing hexadecane (HD), and Hao et al.^15^ followed the same procedure to encapsulate paraffin wax Rubitherm RT25 into a calcium alginate shell.

Although all of these microfluidic devices offer reliable encapsulation and uniform droplets, they are difficult to fabricate and lack the operational flexibility needed to encapsulate a diverse variety of PCMs. In traditional disposable glass capillary devices, needles and capillaries are permanently glued on a microscope glass slide using epoxy glue, and hence readjustment of inner capillaries during the microfluidic process is not possible.^14,16^ This restricts bespoke tuning of the microcapsule structure. In addition, fabrication of traditional glass capillary devices is cumbersome since a minor error in capillary alignment or positioning makes the entire device unserviceable. On the other hand, PDMS channels are sensitive to swelling by organic liquids and their fabrication relies on photolithographically defined silicon master molds, which are very expensive to manufacture.^17^

In this paper, we report a Lego-inspired microfluidic device for the generation of double emulsion droplets and simultaneous on-the-fly UV polymerization to form PCM microcapsules, allowing precise control of PCM loading and particle architecture. The device is composed of coaxial glass capillaries and computer numerical control (CNC)-milled blocks that can be connected and taken apart using a Lego-inspired Stud-and-Tube system. The effectiveness of the new device was shown by the robust encapsulation of PCMs using a Norland Optical Adhesive (NOA), a cheap yet unexplored UV-curable shell material. This nonacrylate photopolymer cured via the thiol-ene “click” reaction was chosen due to the stringent environmental regulations related to acrylates and formaldehyde-based (melamine-formaldehyde/urea-formaldehyde) resins. Two different PCMs, i.e., hexadecane (HD, an organic PCM) and salt hydrate (SH) SP21EK (an aqueous PCM), were chosen due to their high melting enthalpies of 240 ± 2 and 142± 2 J/g, respectively, at a phase change temperature of 20 ± 2 °C. A high degree of control over PCM loading, microcapsule diameter, and the thickness of the microcapsule shell was demonstrated to underline the versatility of the device for the very first time. Lastly, we developed an easy technique to incorporate TiO_2_ nanoparticles (NPs) in the shell and produce multifunctional PCM microcapsules, which concurrently show thermal energy storage and photocatalysis abilities.

## 2 Experimental Section

### 2.1 Materials

For the synthesis of microcapsules, a UV-curable polymer, Norland Optical Adhesive (NOA 81, Norland Products Inc., New Jersey, curing time 10 s), was used as a shell material. NOA is a proprietary adhesive constituted of triallyl isocyanurate and mercapto-ester. To reduce its viscosity, it was diluted with acetone (Sigma-Aldrich) in an NOA:acetone mass ratio of 80:20 and utilized as a middle phase. An organic PCM, hexadecane (*T*_m_ ∼ 20 °C, 99% purity, Sigma-Aldrich), and an inorganic PCM, SP21EK, a commercial salt hydrate (SH) product, probably based on calcium chloride hexahydrate (*T*_m_ ∼ 21 °C, Rubitherm Technologies GmbH, Germany) were used as core materials (inner phases). The outer phase was an aqueous solution of 30 wt % glycerol (Alfa Aesar, 99+ % purity) and 2 wt % poly(vinyl alcohol) (PVA) (31,000–50,000 mol/g, Sigma-Aldrich). Glycerol was used for viscosity adjustment and PVA was the stabilizer for double emulsion droplets. Titanium dioxide nanopowder (anatase TiO_2_, Kronos vlp 7000) was chosen as a photocatalyst and was dispersed in the middle phase in some formulations. All chemicals were used without further purification. The experimental conditions used for the preparation of various samples of PCM microcapsules are shown in Table 1.

**Table 1:** Flow Rates of Phases and Capillary Tip Diameters Used for Preparing Various PCM Microcapsule Samples

	flow rate of phase (mL/h)			capillary tip diameter (μm)	
sample	inner (*Q*_i_)	middle (*Q*_m_)	outer (*Q*_o_)	injection (*D*_ii_)	collection (*D*_ci_)
HD-MC1	1.5	1.5	20	50	200
HD-MC2	1.5	1.5	20	100	400
HD-MC3	1.5	1.5	20	200	500
HD-MC4	0.75	1.5	20	100	400
HD-MC5	3.0	1.5	20	100	400
HD-MC-TiO_2_	3.0	1.5	20	100	400
SP21EK-MC	1.5	1.5	20	100	400

### 2.2 Experimental Equipment and Procedure

#### 2.2.1 Capillaries

Borosilicate round capillaries (World Precision Instruments, U.K.) with ID/OD 1.56/2.0 and 0.58/1.0 mm were used as outer and inner capillaries, respectively. The inner capillary tube was pulled from the middle using a P-97 micropipette puller (Sutter Instruments) and separated into two capillaries, i.e., injection capillary and collection capillary. The tips of both capillaries were adjusted to the desired orifice diameter by grazing over a sandpaper. The orifice diameters were measured using an MF-830 Microforge (Narishige).

#### 2.2.2 Lego Blocks Fabrication

Two interlocking Lego blocks of polyoxymethylene (density 1410 kg/m^3^) were devised using SolidWorks program (Dassault Systèmes) and fabricated using an automated CNC milling machine (HAAS Automation, model Super Mini, Norwich, U.K.). The design of Lego blocks and the device are shown in Figures S1 and S2.

#### 2.2.3 Microfluidic Test Rig

**Figure 1:**
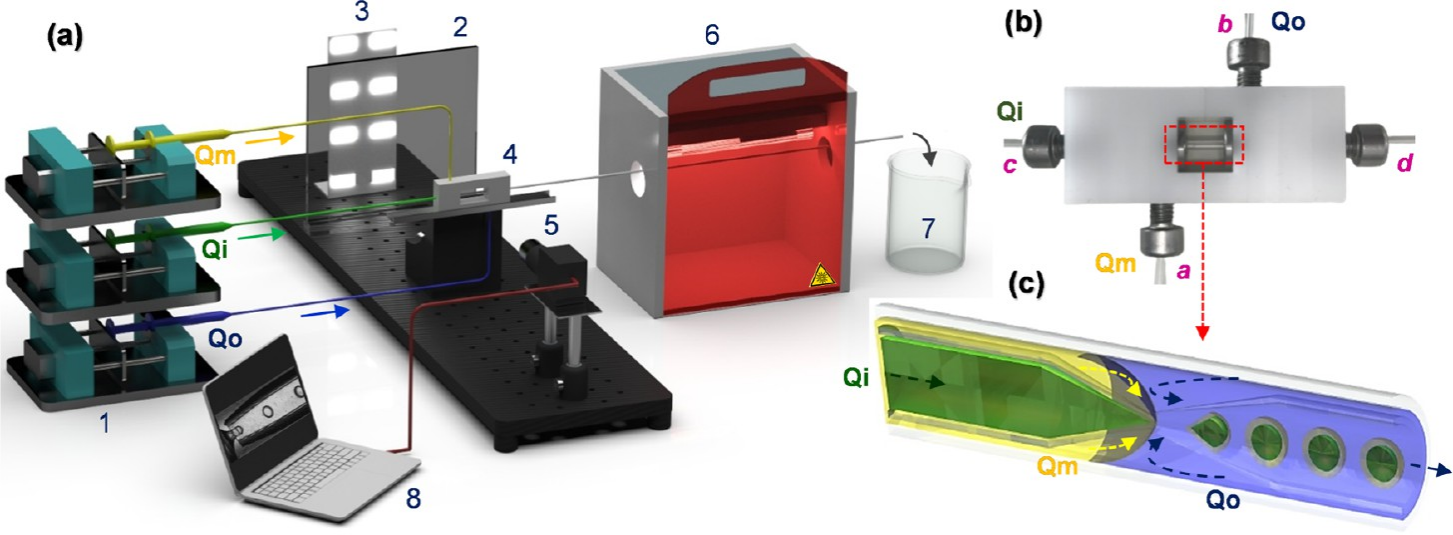
(a) Schematic diagram of the microfluidic test rig setup (1: syringe pumps, 2: light diffuser, 3: LED lights, 4: Lego microfluidic device, 5: camera, 6: UV light chamber, 7: microcapsule collection, 8: computer); (b) Lego microfluidic device with stainless steel connectors (*a*, *b*—medical tubing, *c*, *d*—glass capillaries); and (c) schematic cross-sectional view of core–shell droplet formation.

A bespoke reconfigurable rig was set up for easy and steady production of PCM microcapsules (Figures 1a and S3). The Lego device was placed on the holder mounted on a linear translation stage that can be moved precisely using a micrometer to bring the capillaries into focus. The monochrome camera (DMK 33UX287) equipped with a TMN 1.0/50 lens (1×) was used to observe the droplet generation process. To capture high-resolution images, an LED light panel (48 LED lights, 12 V) and a light-diffusing acrylic sheet were mounted behind the device. The inlets of the middle and outer phases in the device (inlets *a* and *b* in Figure 1b) were closed by metal connectors attached to polyethylene tubing (ID/OD 0.86/1.52 mm, Smiths Medical, U.K.). The tubings were prefilled before connecting to the device to prevent air bubbles. The inner capillaries were tightened in their respective blocks by screwing metal connectors with O-rings in inlets *c* and *d*. The liquid phases were pumped using syringe pumps (Harvard Apparatus 11 Elite, U.K.) from 10, 25, and 50 mL SGE glass syringes (Sigma-Aldrich, U.K.). The microfluidic process was recorded using the IC Capture (V2.5, Imaging Source GmbH, Germany) software and the movie editing and particle size distribution analysis were carried out using the ImageJ program.

Core–shell droplets were generated by counter-current flow focusing of a coaxial jet consisting of PCM and NOA streams (Figure S4). Flow rates were chosen to maximize the loading of PCM while maintaining a stable production of droplets. Core–shell droplets formed in the collection capillary were passed through a UV chamber (45 cm × 20 cm × 25 cm) in a 50-cm-long polyethylene tube to be polymerized “on-the-fly” using two parallel tubular UV lamps (8 W, 220 V) emitting UV-A radiation. The solid microcapsules were collected at the outlet of the UV chamber, washed several times in order to remove PVA and other impurities, and allowed to dry naturally. A high-quality free-flowing final product is shown in Movie S1.

### 2.3 Characterization of Microcapsules

Morphological imaging of microcapsules was performed using scanning electron microscopy (FEG SEM Jeol 7100) equipped with a hot Schottky field-emission gun. An electron beam operated at 7–10 kV was directed onto the sample surface through a vacuum chamber. Energy dispersive X-ray (EDX) spectroscopy with a silicon nitride drift detector was used in combination with SEM for elemental analysis of the shell. Prior to SEM and EDX analysis, all samples were gold-sputtered under a high vacuum.

Thermal energy storage performance was determined using a differential scanning calorimeter (TA Instruments, model Q20) with a heating/cooling rate of 5 °C/min under a nitrogen atmosphere. The thermal cycle span was kept from 0 to 30 and −15 to 60 °C for HD- and SH-based microcapsules, respectively. Thermal stability of microcapsules was investigated using a thermogravimetric analyzer (Mettler Toledo TGA/DSC 1 STAR system). A sample was taken in a platinum pan and placed in a furnace. The temperature of the furnace was raised from 35 to 700 °C in a nitrogen environment at a constant rate of 10 °C/min.

Thermal recyclability of the microcapsules was investigated through thermo-optical observation of the phase transition using a polarizing microscope (Eclipse LV100ND, Nikon, Shinagawa, Tokyo, Japan). The temperature was altered with a temperature-controlled stage (LTS 420, Linkam Scientific, Tadworth, U.K.). The polarizing microscope allows for visualizing phase transitions in the sample via the color change. Samples were embedded in a mucoadhesive Mowiol (Carl Roth, Karlsruhe, Germany) to prevent the movement of the capsules during heating and cooling. To investigate the thermal reliability of microcapsules, each sample was subjected to 100 crystallization–melting cycles in the temperature range of 5–30 and 0–40 °C for HD and SP21EK-based microcapsules at constant cooling/heating rates of 50 °C/min. In-between, the temperature was held constant for 20 s, allowing all capsules to crystallize/melt. For each cycle, one picture was taken in solid and liquid states.

The mechanical properties of the produced microcapsules were investigated using a micromanipulation technique based on the procedure explained elsewhere.^18^ Briefly, the technique was based on compression of a single microcapsule using a borosilicate glass probe (ID/OD 0.58/1.0 mm). The glass probe position was adjusted using a high-resolution micromanipulator (±0.2 μm) and the compression speed of 20.0 μm/s was kept constant to compress 30 capsules of each sample against a fixed tempered-glass surface. A side-view camera (10×, DinoEye C-Mount Camera, U.K.) was mounted to record the process. The compression process was carried out under a semiautomatic mode, which allowed the probe to come back to its original position after the completion of compression.

Photocatalytic performance of microcapsules with TiO_2_-embedded shells was investigated by monitoring the degradation of methylene blue (MB) dye under UV light.^19^ Initially, 1 g of microcapsules was dispersed in 100 mL of 1 ppm MB solution in a glass beaker and placed in a dark room to achieve an adsorption equilibrium. The suspension was then exposed to UV irradiation for 1 h. During this period, 1 mL of the solution was withdrawn every 15 min and its absorbance was measured at a wavelength of 665 nm using a UV–visible spectrophotometer (Thermo Scientific NanoDrop One C). The rate of degradation (*D*) was calculated using the equation
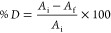
1where *A*_i_ and *A*_f_ are the initial and final absorbance, respectively.

## 3 Results and Discussion

### 3.1 Synthesis Strategy of Microcapsules

The Lego-inspired microfluidic device is shown in Figure 1b. The device consists of two tapered-end round capillaries arranged with the tips facing each other in one bigger round capillary. The inner diameter of the big capillary (1.56 mm) is slightly larger than the outer diameter of the inner capillaries (1 mm). In comparison to conventional glass capillary devices, where round capillaries are arranged in an outer square capillary, this configuration allows for round-inside-round capillary geometry.^20,21^ The main benefit of the Lego device is the reduction of the setup time from 1 h to several minutes. Additionally, the capillaries can be cleaned and reused in case of a blockage. The inner PCM phase was delivered through the left injection capillary. The middle NOA polymer phase was injected through the annular space between the injection capillary and the big capillary. The outer phase was injected from the opposite side through the annular space between the collection capillary and the big capillary. The NOA middle phase encapsulates the inner PCM droplet as it leaves the injection capillary, and core–shell droplets are formed in the entry region of the collection capillary. The droplet generation rate was 3350 drops/min (Figure 1c).

The droplets remained stable until the polymerization was completed only when a polymeric PVA surfactant was used. In contrast, short-chain surfactants such as polysorbate 20 (Tween 20) and sodium lauryl sulfate (SLS) were not effective in stabilizing the droplets. It seems that the steric hindrance of the polymer chains adsorbed at the liquid interface can more efficiently prevent contact of the interfaces and therefore inhibit the breakdown of the liquid NOA shells before polymerization.^22^ This is in accordance with our previous works showing that PVA is a superior stabilizer for polymeric droplets generated in microfluidic devices.^23,24^

**Figure 2:**
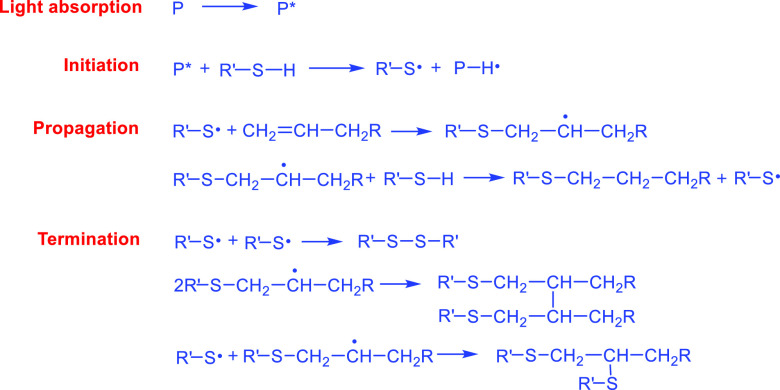
Schematic showing the plausible reaction of thiol-ene UV-initiated polymerization by a radical step-growth mechanism.

NOA is cured via the reaction between a mercapto-ester and triallyl isocyanurate that follows thiol-ene click chemistry for photoinitiated polymerization.^25^ Unlike chain propagation in acrylic monomers, it proceeds through a radical step-growth mechanism (Figure 2).

First, the photoinitiator (P) dissociates under UV light and forms an excited species (P*). P* combines with the thiol group by hydrogen abstraction and produces the thiyl radical (R′–S^•^). Second, the thiyl radical addition to the allyl group forms the thioether carbon radical (R′–S–CH_2_–ĊH–CH_2_R) as an intermediate step. It then generates an additional thiyl radical via hydrogen abstraction, thus propagating the polymerization via an anti-Markovnikov addition. Due to the presence of oxygen, it produces a peroxy radical, which causes a chain-transfer reaction with thiol and gives an oxygen addition product and propagating thiyl radical. Lastly, the termination of the reaction happens by recombination between the thioether carbon and thiyl radicals.^26^

### 3.2 Droplet Generation Regimes

The drop breakup in capillary devices can proceed in two distinct regimes: (i) dripping regime, due to absolute jet instability in which jet perturbations cause drop pinch-off at a fixed downstream location and with a constant frequency determined by the properties of the system, forming monodisperse droplets and (ii) jetting regime, where drop generation is a result of convective instability, which is caused by slight imperfections of the laminar flow at the pinch-off. These imperfections lead to perturbations at the interface that evokes oscillations leading to drop breakup. As a result of this breakup mechanism, polydisperse droplets form at varying breakup points.^24^ The drop formation mechanisms are dictated by a trade-off between inertial, viscous, interfacial, and gravitational forces.^10,27^ These forces are affected by the physical properties of the phases presented in Table 2.

**Table 2:** Physical and Interfacial Properties of the Inner, Middle, and Outer Phases for HD-Based Microcapsules at 25 °C

phase	viscosity μ (Pa·s)	density ρ (kg/m^3^)	interfacial tension σ (mN/m)
inner phase (HD)	0.0033	773	σ_im_ = 6.3
middle phase (80% NOA + 20% acetone)	0.0119	1176	σ_mo_ = 9.2
outer phase (30% glycerol + 2% PVA + 68% water)	0.0047	1078	σ_oi_ = 49.8

**Figure 3:**
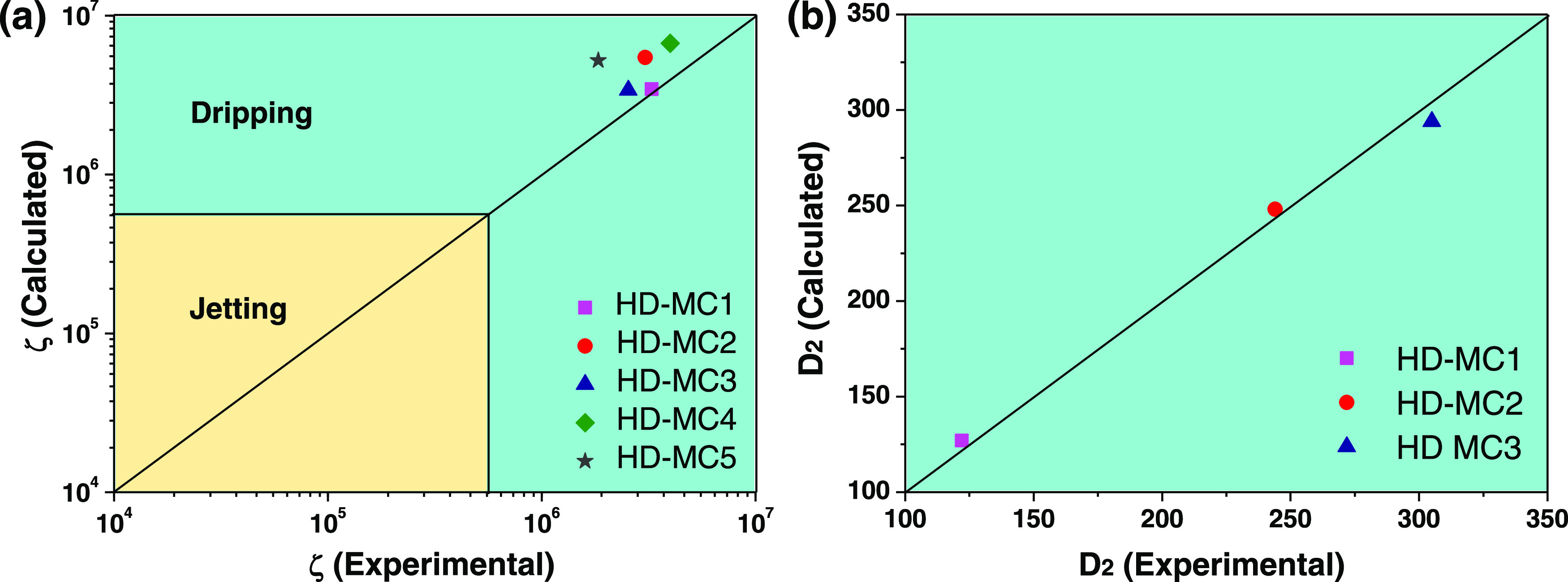
(a) Comparison between the experimental ζ values determined from eq 4 and the ζ values calculated from eq 3 for samples of HD capsules in Table 1. The dripping regime occurs at ζ > 5 × 10^5^, while jetting prevails at ζ < 5 × 10^5^. (b) Comparison between the *D*_2_ values calculated from eqs 5 and 6 and the experimental *D*_2_ values.

Operating a microfluidic device within the dripping regime has remained a major challenge. The mode of drop formation can be adjusted by changing the fluid properties (e.g., viscosity and interfacial tension) and the flow rates that will affect the capillary number of inner phase (*Ca*_i_), middle phase (*Ca*_m_), and outer phase (*Ca*_o_)
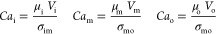
2where *V*_i_ = 4*Q*_i_/(π*D*_*i*_^2^), *V*_m_ = 4*Q*_m_/[π(*D*_bi_^2^ – *D*_io_^2^)], and *V*_o_ = 4*Q*_o_/[π(*D*_bi_^2^ – *D*_co_^2^)]. Here, *V*_i_, *V*_m_, and *V*_o_ stand for velocities of inner, middle, and outer phases, respectively, μ_i_, μ_m_, and μ_o_ are viscosities of these fluids, *D*_bi_ is the ID of the big (outer) capillary tube, and *D*_io_ is the OD of the inner capillary tube (Figure S4). The drop formation regime also depends on the orifice diameters, with higher *D*_ci_/*D*_ii_ values favoring the dripping mode. These parameters can be grouped in a single dimensionless parameter
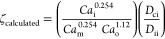
3One property that shows large variations depending on the drop generation regime is *D*_2_/*L*, where *D*_2_ is the outer droplet diameter and *L* is the breakup length (distance between the orifice tip of the injection capillary and the location of drop pinch-off).^27^ In the dripping regime, *D*_2_/*L* is close to unity, while in the jetting regime it has a much smaller value. The parameter ζ defined by eq 3 can be determined from experimental *D*_2_/*L* values using eq 4(27)
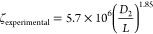
4In Figure 3a, we tested if ζ can be applied to predict the exact regime of drop formation within our Lego device. In traditional round-in-square capillary geometry,^27^ double emulsion droplets form in the dripping mode when log ζ > 5.7, i.e., ζ > 5 × 10^5^, while the jetting mode is prevalent for log ζ < 5.7. The data points corresponding to five samples of HD capsules in Table 1 are relatively close to the diagonal line, which confirms a good match between the ζ values determined from eqs 4 and 3. All ζ values are much higher than the critical ζ value, verifying that core–shell droplets were indeed formed in the dripping regime.

We expanded our investigation to elucidate whether droplet diameters *D*_2_ could be predicted from fluid flow rates. In the dripping regime, the velocities of all fluid streams at the inlet of the collection capillary are approximately the same,^10^ which means that the flow rates are proportional to the corresponding cross-sectional areas
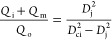
5where *D*_j_ is the diameter of a compound jet composed of two coaxial liquid streams, which can be related to the resultant droplet diameter as follows
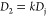
6where *k* is the proportionality constant that is a function of the maximum jet instability (*in*): *k* = (3π/2*in*)^1/3^. The maximum jet instability depends on the viscosity ratio (μ_effective_/μ_o_), where μ_effective_ is the effective jet viscosity of the mixture of inner and middle phases and μ_o_ is the outer phase viscosity. Since *Q*_i_/*Q*_m_ remains unchanged for the samples HD-MC1, HD-MC2, and HD-MC3, the viscosity ratio μ_effective_/μ_o_ becomes constant.^10,28^ Therefore, these three samples can be used to test eq 6. Comparison of the experimental *D*_2_ and *D*_j_ values for samples HD-MC1, HD-MC2, and HD-MC3 using eq 6 showed that *k* ≈ 1.69. The calculated *k* value differs from 1.87 found by Utada et al.,^10^ which is probably due to different phases used in their study. As shown in Figure 3b, the *D*_2_ values calculated using eqs 5 and 6 are in excellent correspondence with the experimental *D*_2_ values.

### 3.3 Morphology Control of Microcapsules

**Figure 4:**
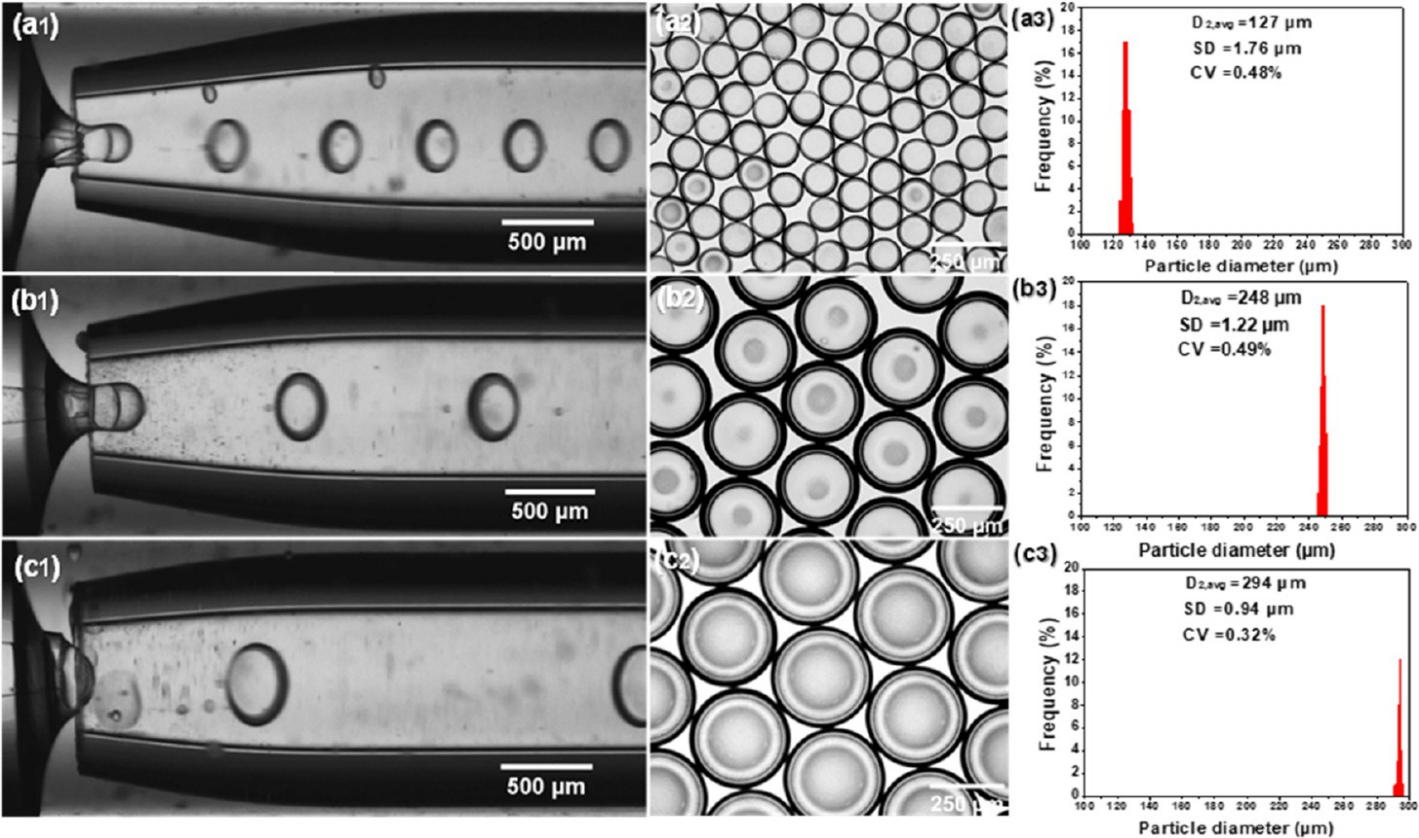
Variation of particle diameters of HD-loaded microcapsules at constant flow rates of *Q*_i_ = 1.5 mL/h, *Q*_m_ = 1.5 mL/h, and *Q*_o_ = 20 mL/h: (a1) *D*_ii_ = 50 μm and *D*_ci_ = 200 μm (HD-MC1); (b1) *D*_ii_ = 100 μm and *D*_ci_ = 400 μm (HD-MC2); (c1) *D*_ii_ = 200 μm and *D*_ci_ = 500 μm (HD-MC3). (a2, b2, c2) Bright-field microscopy images of the generated microcapsules and (a3, b3, c3) are their size distributions, respectively.

To confirm that the size of PCM microcapsules can be controlled by adjusting the orifice diameter of the injection and collection capillary tubes, HD-loaded microcapsules were produced at constant flow rates (*Q*_i_ = 1.5 mL/h, *Q*_m_ = 1.5 mL/h, and *Q*_o_ = 20 mL/h), but the orifice diameters were varied from *D*_ii_ = 50 and *D*_ci_ = 250 μm (MC-HD1) to *D*_ii_ = 100 and *D*_ci_ = 400 μm (MC-HD2), and to *D*_ii_ = 200 and *D*_ci_ = 500 μm (MC-HD3). The produced droplets and microcapsules and their size distributions are shown in Figure 4 and Movie S2. It should be noted that droplets in the collection capillary appear elliptical due to optical distortion caused by the cylindrical surface of the outer capillary.

The uniformity of the microcapsules was determined by calculating the coefficient of variation (CV)
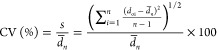
7where *s* is the standard deviation of particle sizes, *d*_oi_ is the outside diameter of the *i*th particle, and *d̅*_*n*_ is the number-average outside diameter. When increasing the orifice diameter of the collection capillary (*D*_ci_) from 200 to 500 μm, the mean diameter of the capsules increased from 127 to 294 μm, confirming the trend observed earlier.^29^ When *D*_ii_ and *D*_ci_ had the smallest values of 50 and 200 μm (HD-MC1), the PCM microcapsules had the average diameter of 127 μm (Figure 4a3). Since *Q*_i_, *Q*_m_, and *Q*_o_ were kept constant, under these conditions, the flow velocity of the outer phase at the entrance of the collection capillary had the maximum value, which caused the highest drag force at the interface between the outer and middle phases and the jet had the maximum velocity and minimum thickness. As a result, smallest droplets were produced at the highest frequency, which is apparent by the smallest spacing between the neighboring droplets in Figure 4a1. The same conclusion can be drawn from Movie S2. For the higher *D*_ii_ and *D*_ci_ values of 100 and 400 μm (HD-MC2), the average diameter of microcapsules was 248 μm (Figure 4b3). When the orifice diameters (*D*_ii_ and *D*_ci_) increased to the final values of 200 and 400 μm (HD-MC3), the average particle diameter was further increased to 294 μm (Figure 4c3). The CV values are 0.32–0.49%, thus much less than 3%, which is considered as the maximum CV value for monodispersed particles. Monodispersed PCM microcapsules are preferred since they exhibit uniform heat storage capacity and uniform heat transfer behavior and mechanical properties. Also, monodisperse particles offer minimum resistance to fluid flow if they form a fixed bed on the solid substrate.

**Figure 5:**
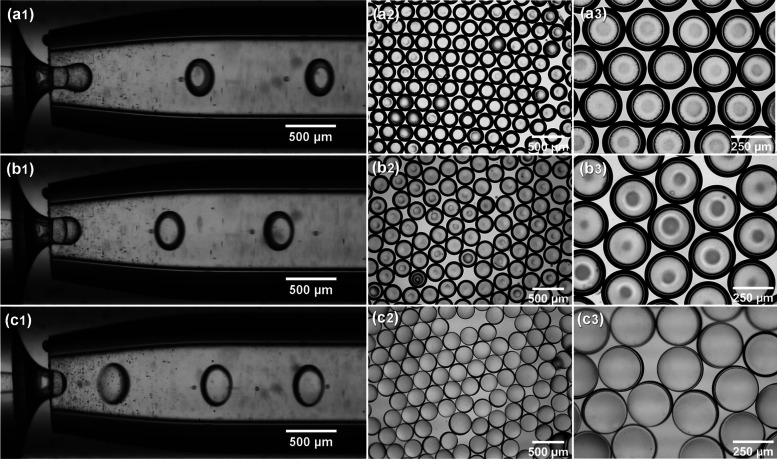
Formation of core–shell droplets with different shell thicknesses at *D*_ii_ = 100 μm, *D*_ci_ = 400 μm, and flow rates: (a1) *Q*_i_ = 0.75 mL/h, *Q*_m_ = 1.5 mL/h, *Q*_o_ =2 0 mL/h (HD-MC4); (b1) *Q*_i_ = 1.5 mL/h, *Q*_m_ = 1.5 mL/h, *Q*_o_ = 20 mL/h (HD-MC2); (c1) *Q*_i_ = 3 mL/h, *Q*_m_ = 1.5 mL/h, *Q*_o_ = 20 mL/h (HD-MC5). (a2, b2, c2) and (a3, b3, c3) are the corresponding bright-field microscopy images of the generated microcapsules at 4× and 10× magnifications.

Next, we sought to understand the effect of the PCM flow rate (*Q*_i_) on the shell thickness of the microcapsules. An increased thickness of the shell is an easy way to increase the thermal and mechanical stabilities of PCM microcapsules. Thin shells may be broken under mechanical or thermal stress and cause leakage of the PCM, thereby reducing the product durability. However, excessive shell thickness decreases the PCM loading and heat storage capacity of the microcapsules. The shell thickness of core–shell droplets with an outer diameter *d*_o_ can be predicted by the mass balance equation of the droplet formation process
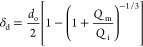
8For a constant orifice size, the droplet diameter *d*_o_ mainly depends on *Q*_o_ and *Q*_m_. Therefore, for constant *Q*_m_ and *Q*_o_, the capsule size will be mostly unaffected, and the shell thickness can be controlled by changing *Q*_i_, with higher *Q*_i_ values leading to thinner shells according to eq 8. To demonstrate this feature, *Q*_i_ was varied from 0.75 mL/h (HD-MC4) to 1.5 mL/h (HD-MC2) to 3 mL/h (HD-MC5), while keeping *Q*_m_ = 1.5 mL/h, *Q*_o_ = 20 mL/h, *D*_ii_ = 100 μm, and *D*_ci_ = 400 μm constant. The bright-field micrographs of the resulting microcapsules shown in Figure 5 confirm that *D*_2_ remained nearly the same (250–280 μm), but the shell thickness was significantly smaller at higher *Q*_i_ values, as predicted by eq 8.

**Figure 6:**
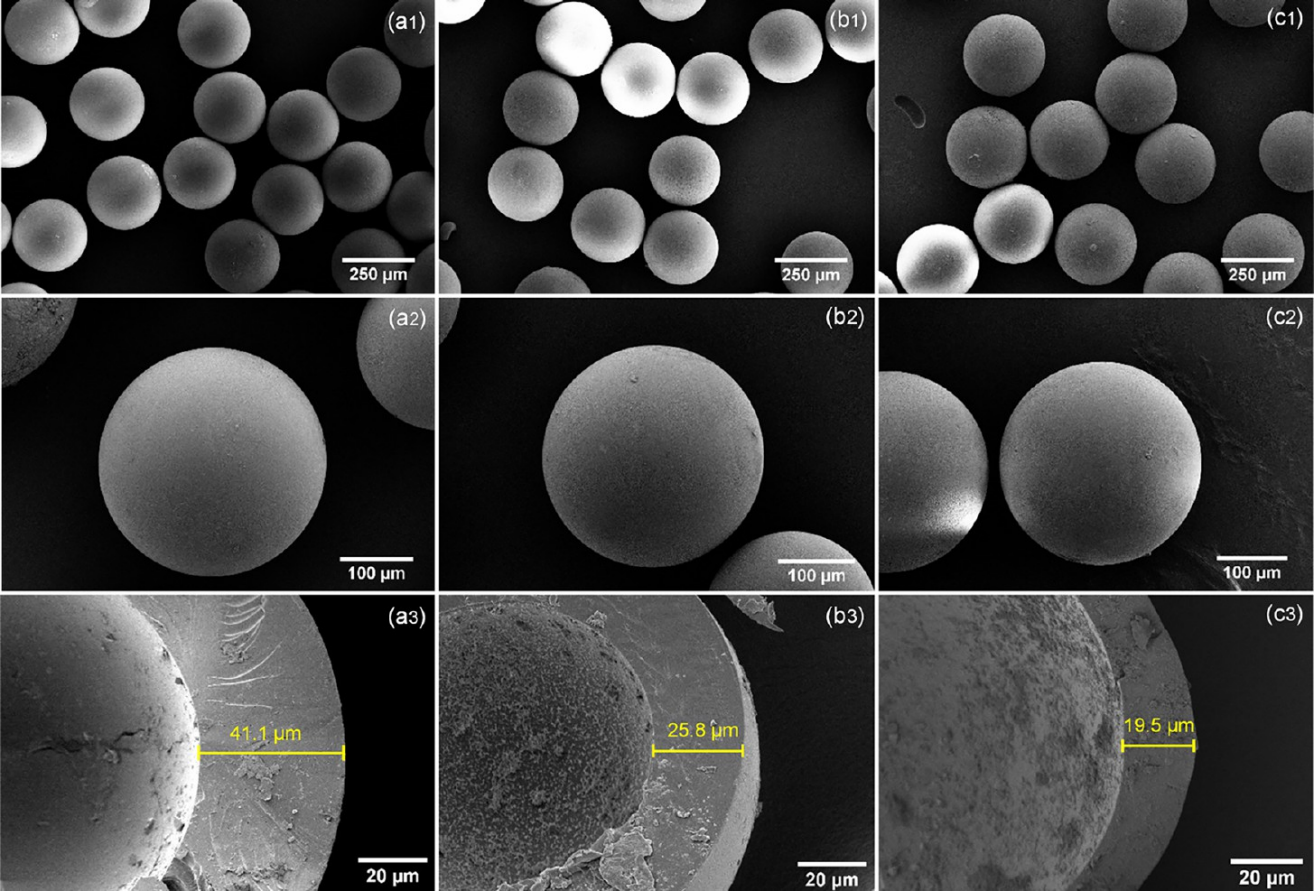
SEM micrographs of HD-loaded microcapsules at different magnifications prepared at orifice diameters *D*_ii_ = 100 μm and *D*_ci_ = 400 μm and flow rates: (a1, a2) *Q*_i_ = 0.75 mL/h, *Q*_m_ = 1.5 mL/h, *Q*_o_ = 20 mL/h (HD-MC4); (b1, b2) *Q*_i_ = 1.5 mL/h, *Q*_m_ = 1.5 mL/h, *Q*_o_ = 20 mL/h (HD-MC2); (c1, c2) *Q*_i_ = 3 mL/h, *Q*_m_ = 1.5 mL/h, *Q*_o_ = 20 mL/h (HD-MC5); and (a3, b3, c3) corresponding shell thicknesses after microtome cutting.

SEM was used to study the surface morphology of the microcapsules, while their shell thicknesses were accurately measured using SEM microtomy. Surface morphology is closely related to the dispersibility, flowability, adherence behavior, and attrition resistance of particles and is therefore essential in the quality assessment of the PCM microcapsules. As shown in the SEM images in Figure 6, the prepared microcapsules had a regular spherical shape with a narrow particle size distribution and without any interparticle bridges, which can be attributed to the robust emulsification process and rapid on-the-fly polymerization. From the magnified SEM images in Figure 6a2, b2, c2 and optical microscopy images (Figure S5), it can be seen that the microcapsules exhibit a smooth and compact surface without dimples despite the high vacuum applied during the preparation of samples. No surface impurities or clustered particles were found, confirming the shell material was completely polymerized. In addition, the microcapsules can flow freely, with no sign of stickiness due to particle–particle adhesion (Movie S1).

The shell thickness of microcapsules measured after microtome cutting decreased from 41.1 to 25.8 to 19.5 μm in Figure 6a3,b3,c3 and was similar to the values predicted from the mass balance of the microfluidic process. The smallest shell thickness of 19.5 μm (Figure 6c3) was achieved at the highest inner phase flow rate of 3 mL/h corresponding to the *Q*_i_/*Q*_m_ ratio of 2. With a further increase in acetone content in the NOA phase and a fine adjustment of fluid flow rates, we believe that the shell thickness can be reduced below 19 μm and subsequently, PCM loading can be further improved. Close inspection of the cross section of the dried microcapsules in Figure 6a3,b3,c3 revealed a dense polymeric shell, indicating that evaporation of acetone from the middle phase did not cause any porosity within the NOA shell.

Movie S3 shows the dependence of the shell thickness on the inner phase flow rate, which is in good agreement with previous studies on similar double emulsion systems.^30,31^ This relationship can also be understood from the basic mass balance equations of the process. The volume flow rate of the core material (HD) is
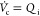
9The volume flow rate of pure NOA (shell-forming material) is
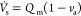
10where *v*_a_ is the volume fraction of acetone in the middle phase given by
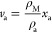
11where ρ_M_ and ρ_a_ are the densities of the middle phase (NOA/acetone mixture) and pure acetone, respectively, and *x*_a_ is the weight fraction of acetone (0.2). The volume fraction of HD in microcapsules is given by
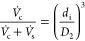
12By combining eqs 9, 10, and 12, the core diameter (*d*_i_) of microcapsules can be calculated based on the capsule diameter *D*_2_, flow rates *Q*_i_ and *Q*_m_, and volume fraction of acetone
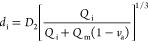
13The shell thickness of particles (δ_p_) can be then calculated using the equation
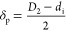
14The shell thicknesses of HD-MC4, HD-MC2, and HD-MC5 calculated from eq 14 are 40.1, 25.2, and 17.5 μm, respectively, which agrees well with the values obtained from SEM images of cross-sectioned microcapsules. This verifies that HD was completely enclosed within the microcapsules and the presented mass balance equations can be used to estimate the required flow rates to accurately tune the thickness of HD microcapsules.

Lastly, HD as an inner phase was replaced with salt hydrate SP21EK, while keeping other experimental conditions the same as for HD-MC2. The encapsulation of salt hydrate PCMs (aqueous PCMs) is considered highly challenging due to their hydrophilic nature and poor chemical compatibility with most polymers.^32^ Traditional fabrication methods for encapsulating hydrated salts are based on suspension polymerization, sol–gel process followed by interfacial polymerization, and interfacial polymerization.^33^ These processes require over 6 h cycle time and the obtained capsules are highly polydisperse with a low encapsulation efficiency. Herein, for the first time, we report microfluidic encapsulation of salt hydrate SP21EK in NOA. As shown in the SEM image in Figure S6b, SP21EK microcapsules are compact with a regular spherical shape and smooth surface, which indicates that SP21EK was successfully encapsulated in NOA and PVA provided a good stabilization of NOA droplets against coalescence. Figure S6c confirms that a core–shell structure was formed during the encapsulation process and the shell thickness of microcapsules was 26.4 μm. The synthesized microcapsules were found to be highly monodisperse with diameters distributed in a narrow range of 250–260 μm.

### 3.4 Thermal Energy Storage Performance of Microcapsules

**Figure 7:**
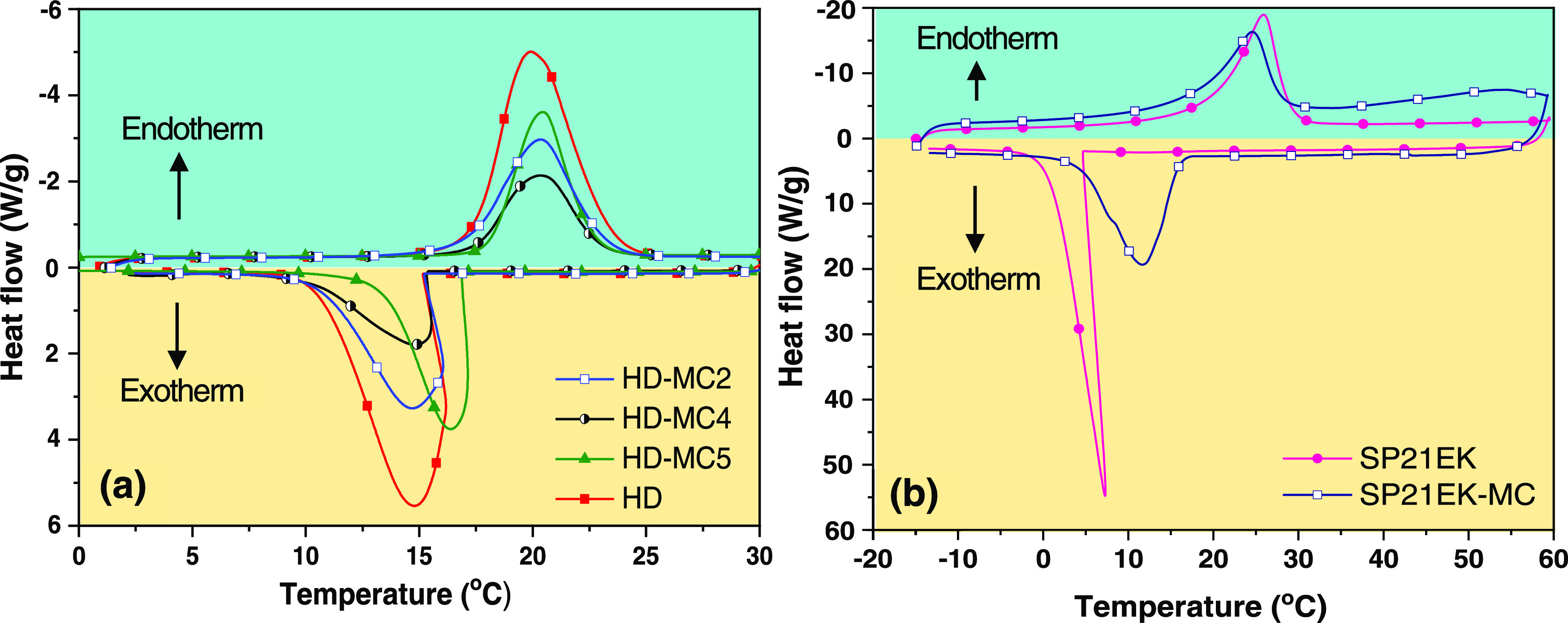
DSC thermogram of (a) pure HD, HD-MC2, HD-MC4, and HD-MC5 and (b) pure SP21EK and SP21EK-MC.

High melting enthalpy is the main requirement for an efficient PCM. The thermal energy storage properties of PCM microcapsules can be readily predicted using mass balance equations of the microfluidic process. The theoretical PCM content or encapsulation efficiency *E*_theo_ of the microcapsules enclosing HD can be calculated from the mass flow rates (in g/h) of HD (*ṁ*_h_), NOA (*ṁ*_NOA_), and acetone (*ṁ*_a_)
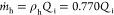
15
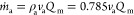
16
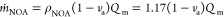
17where ρ_h_, ρ_a_, and ρ_NOA_ are the densities (in g/mL) of HD, acetone, and NOA, respectively; see Table 2. If all acetone is diffused into the outer phase and then evaporated into ambient air, the theoretical encapsulation efficiency of HD in the microcapsules is given by
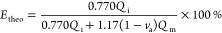
18All of the microcapsule samples enclosing HD and SP21EK, as well as pure PCMs, were analyzed using differential scanning calorimetry (DSC) to compare the experimental and theoretical encapsulation efficiencies of the PCM and estimate their thermal properties. The obtained DSC thermograms are shown in Figure 7a,b and the thermal properties of the materials are summarized in Table 3.

**Table 3:** Thermal Properties of Pure PCMs (HD and SP21EK) and PCM Microcapsules (*T*_m_ = Melting Temperature, Δ*H*_m_ = Melting Enthalpy, *T*_c_ = Crystallization Temperature, Δ*H*_c_ = Crystallization Enthalpy, *E* = Encapsulation Efficiency of PCM)

sample	*T*_m_ (°C)	Δ*H*_m_ (J/g)	*T*_c_ (°C)	Δ*H*_c_ (J/g)	*E*_exp_ (%)	*E*_theo_ (%)
HD	19.9	240	14.7	239	NA	NA
HD-MC4	20.4	74	15.0	74	30.8	29.0
HD-MC2	20.3	111	14.9	110	46.2	45.0
HD-MC5	20.3	157	16.4	158	65.4	62.0
SP21EK	25.7	142	7.2	143	NA	NA
SP21EK-MC	24.6	64	12.5	64	44.3	NA

The phase change enthalpies were estimated by calculating the total areas under the peaks obtained during the heating or cooling stage of a DSC cycle. The phase change temperatures were estimated from the peak positions. The theoretical encapsulation efficiencies (*E*_theo_) were calculated from eq 18, while the experimental encapsulation efficiencies (*E*_exp_) were calculated from the enthalpies of pure and encapsulated PCM using the following equation
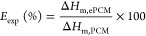
19where Δ*H*_m,ePCM_ and Δ*H*_m,PCM_ denote melting (crystallizing) enthalpies of encapsulated PCM and pure PCM, respectively.

As shown in Figure 7a, the thermogram of pure HD exhibited a remarkable melting enthalpy of 240 J/g with a melting temperature at 19.9 °C, confirming that HD is a good PCM. The thermogram of the corresponding microcapsules (HD-MC4, HD-MC2, and HD-MC5) showed a similar phase change behavior to those of pure HD. Therefore, chemical interactions between PCM and the shell material can be excluded. Also, the intrinsic thermal storage and release properties of HD during phase transition were preserved after encapsulation. Moreover, the melting temperatures of all microcapsule samples were very close to the value of pure HD, which shows that the thermal storage properties indeed come from the encapsulated HD and not from the polymeric shell. Compared with pure HD, the melting temperatures of samples HD-MC4, HD-MC2, and HD-MC5 increased by 0.5, 0.4, and 0.4 °C, respectively, due to the thermal resistance of the shell delaying the phase change of PCM at the applied heating ramp and causing the melting peak to shift toward higher temperatures. It is reasonable to expect that any difference in temperature between the polymeric shell and the core would disappear at very small heating rates and the melting temperatures of encapsulated and pure HD would be the same. The effect of shell thickness on the melting temperature was negligible although the highest increase in melting temperature was observed for the sample with the thickest shell (HD-MC4). Similarly, crystallization temperatures of HD-based microcapsules were also influenced by their shell thicknesses. The pure HD showed a crystallization temperature (*T*_c_) of 14.7 °C, while *T*_c_ for the encapsulated HD samples was higher (15–16.4 °C). Presumably, encapsulation of HD increased the specific surface area, which promoted the heterogeneous crystallization and increased the crystallization temperature as compared to the pure HD. The sample with the lowest shell thickness (19.5 μm), i.e., HD-MC5, exhibited an early crystallization at *T*_c_ = 16.4 °C as compared to HD-MC4 (*T*_c_ = 15 °C) and HD-MC2 (*T*_c_ = 14.9 °C). Lowering the crystallization temperature could be attributed to the higher thermal resistance of thicker HD-MC4 and HD-MC2 shells.

The experimental encapsulation efficiencies of samples HD-MC4, HD-MC2, and HD-MC5 were found to be 30.8, 46.2, and 65.4%, while the theoretical encapsulation efficiencies are 29, 45, and 62%, calculated using basic mass balance equations. The increase in latent heat for the samples prepared at higher *Q*_i_/*Q*_m_ values could be attributed to a higher amount of PCM per polymer injected. A close match between *E*_exp_ and *E*_theo_ values indicates that PCM was fully incorporated within the capsules without any subsequent loss due to evaporation or leakage and that all acetone from the middle phase was removed by diffusion into the continuous phase.

The DSC thermograms of pure and encapsulated SP21EK (denoted as SP21EK and SP21EK-MC) are shown in Figure 7b. The latent heat of pure salt hydrate estimated from the endothermic peak was 142 J/g, while for SP21EK-MC it was 64 J/g, suggesting that the loading of SP21EK in the microcapsules was 44.3%. For pure SP21EK, there was a big difference between *T*_m_ and *T*_c_ with a very sharp and narrow exothermic peak, indicating a significant degree of supercooling followed by very fast crystal nucleation from a highly supercooled melt. The results show that encapsulation significantly affects the supercooling behavior of SP21EK. After encapsulation, the degree of supercooling of SP21EK decreased from 18.5 to 12.1 °C because the large specific surface area of the NOA shell provided numerous nucleation sites for the crystallization of SP21EK in the core. The supercooling suppression can be explained by smaller energy barriers in heterogeneous nucleation. For spherical crystal nuclei, the free energy barrier a nucleus must overcome before further growth is energetically favored is defined as^34^
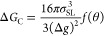
20
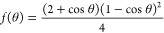
21where σ_SL_ is a solid–liquid interface free energy, θ is the contact angle, and Δ*g* is the melt-crystal free energy difference per unit particle volume. From eqs 20 and 21, θ must be low to reduce Δ*G*_C_ such that for the shell surface completely wetted by PCM (θ = 0), the rate of nucleation is at maximum because Δ*G*_C_ = 0.

Thermal energy storage performance of encapsulated PCMs from this work and previous studies are compared in Table 4. The energy storage performance of the PCM microcapsules synthesized was similar to or even better than previously reported values. Therefore, a Lego device is a highly efficient tool for the encapsulation of both organic and inorganic PCMs.

**Table 4:** Comparison of Energy Storage Performance between Produced Microcapsules and Microcapsules Reported in the Literature

type of PCM	core	shell	encapsulation technique	*E* (%)	ref
organic PCM	caprylic acid	polystyrene	emulsion polymerization	45.4	(35)
	paraffin	polyurea	interfacial polymerization	44.5	(36)
	*n*-docosane	silica	sol–gel	58	(37)
	poly(melamine-urea-formaldehyde)	decanoic acid	*in situ* polymerization	53.9	(38)
	*n*-tetradecane	calcium carbonate	self-assembly	25.86	(39)
	*n*-hexadecyl bromide	elastic silicone	microfluidics	48.4	(13)
	HD	NOA	Lego microfluidics	65.4	this work
inorganic PCM	sodium phosphate dodecahydrate	poly(cellulose acetate butyrate-*co*-methylene diisocyanate)	solvent evaporation precipitation	40.3	(40)
	sodium thiosulfate pentahydrate	poly(ethyl-2-cyanoacrylate)	interfacial polymerization	51.1	(41)
	SP21EK	NOA	Lego microfluidics	44.3	this work

### 3.5 Thermal Stability of Microcapsules

**Figure 8:**
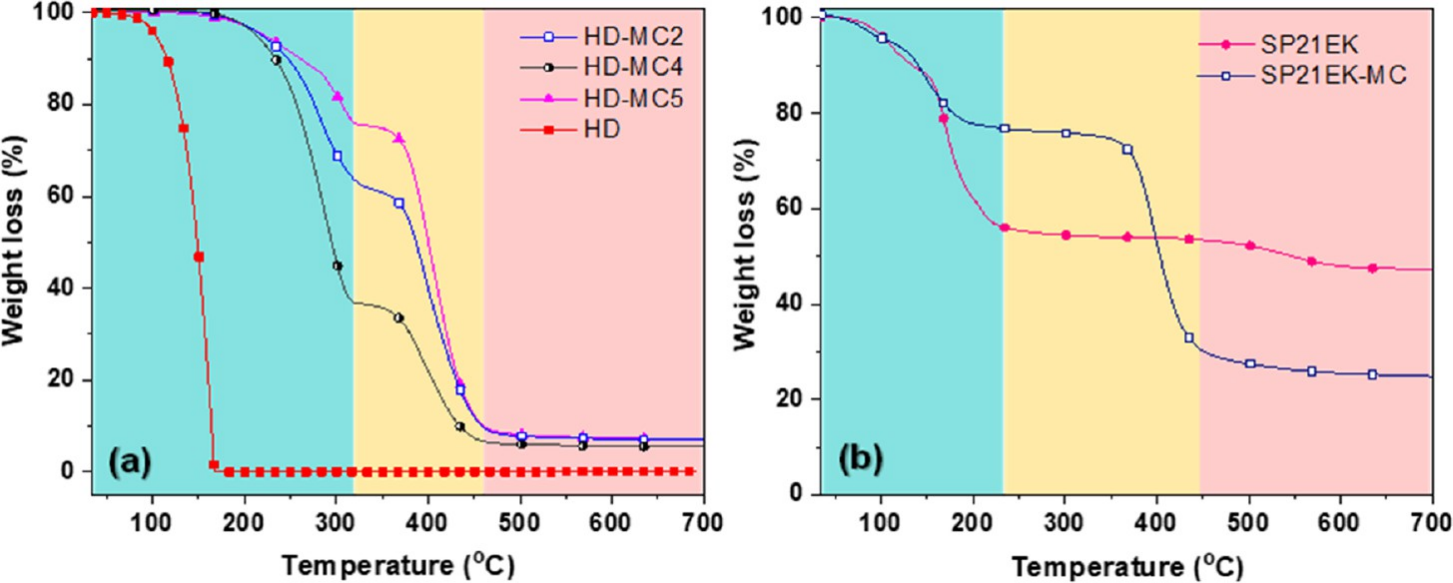
TGA thermograms of (a) pure HD, HD-MC2, HD-MC4, and HD-MC5 and (b) pure SP21EK and SP21EK-MC.

The thermal stability of PCM microcapsules determines their maximum operating temperature and depends on the type of PCM and the shell material. Thus, thermogravimetric analysis was performed to acquire the composition and thermal stability data. The thermograms of microcapsules enclosing HD and SP21EK are presented in Figure 8a,b, respectively. The degradation properties and ash content acquired from the thermogram of both types of microcapsules are summarized in Table 5.

**Table 5:** TGA Data of Pure HD, Pure SP21EK, and All PCM Microcapsule Samples

		first stage		second stage		
sample	onset (°C)	*T* (°C)	weight loss (%)	*T* (°C)	weight loss (%)	residue (%)
HD-MC2	247	247–341	41.8	341–476	51.4	6.8
HD-MC4	262	262–324	25.9	324–473	65.3	7.1
HD-MC5	235	235–341	63.4	341–478	31.0	5.6
HD	116	116–168	100	NA	NA	0.0
SP21EK	110	110–231	55.3	231–496	4.4	40.3
SP21EK-MC	117	117–359	25.9	359–483	49.3	24.8

Figure 8a (HD-based microcapsules) shows that the pure PCM exhibited one-step degradation in the temperature range of 116–168 °C with no residue, indicating complete evaporation of liquid HD. On the other hand, all PCM microcapsules (HD-MC4, HD-MC2, and HD-MC5) revealed three degradation stages. The first stage takes place at ∼230–345 °C and is induced by the swelling of the polymeric shell and loss of PCM due to permeation through the swelled polymer network. The second stage assigned to melting and thermal degradation of the NOA shell due to pyrolysis occurred at ∼320–480 °C. Lastly, the third stage with negligible weight losses occurred at 480–700 °C due to the polymer residue left after complete degradation. The weight losses in the first degradation stage, 25.9, 41.8, and 63.4% for HD-MC4, HD-MC2, and HD-MC5, respectively, were in excellent agreement with the encapsulation efficiencies obtained from the DSC curves (Table 3). The onset temperature for HD evaporation was 116 °C, assuming ∼5% of initial weight loss was due to moisture. The onset degradation temperatures of microcapsules were higher than that of pure HD, indicating their higher thermal stabilities. Therefore, the NOA shell proved to protect HD against the applied temperature and delayed the release of HD. The second-stage weight losses were due to degradation of side groups and backbone bonds of the polymer. As the inner-to-middle phase ratio was altered from 0.5:1 (HD-MC4) to 2:1 (HD-MC5), the shell thickness was reduced from 41.1 to 19.5 μm, as shown in Figure 6c. The reduced shell thickness of HD-MC5 reduced the thermal stability of this sample compared to HD-MC4. Finally, the residual mass left at 700 °C was 7.1% (HD-MC4), 6.8% (HD-MC2), and 5.6% (HD-MC5). A decrease in the residual mass from sample HD-MC4 to HD-MC2 to HD-MC5 reflects smaller and smaller shell content in the respective formulations.

The thermograms of samples SP21EK and SP21EK-MC are shown in Figure 8b. Pure SP21EK began to lose weight at room temperature and completely lost crystalline water molecules to form an anhydrous salt at 231 °C. The total weight loss was 55.3% and could be attributed to PCM dehydration and subsequent water evaporation. The anhydrous salt remained unburnt due to its high melting temperature. SP21EK-MC started to degrade at 117 °C and underwent accelerated mass loss from 359 °C due to thermal decomposition of the shell material. Initially, the weight loss of SP21EK-MC was smaller than that of SP21EK because the NOA shell slowed down dehydration of the hydrated salt. However, in the temperature range of 359–483 °C, the weight loss of SP21EK-MC was 49.3% (due to shell decomposition), as compared to negligible weight loss of SP21EK (since water was already removed and NOA was not present). The residue of SP21EK-MC left at 483 °C was 24.8% and corresponds to anhydrous salt and a part of the NOA shell that remained undecomposed at 700 °C.

### 3.6 Thermal Reliability and Leakage Performance

**Figure 9:**
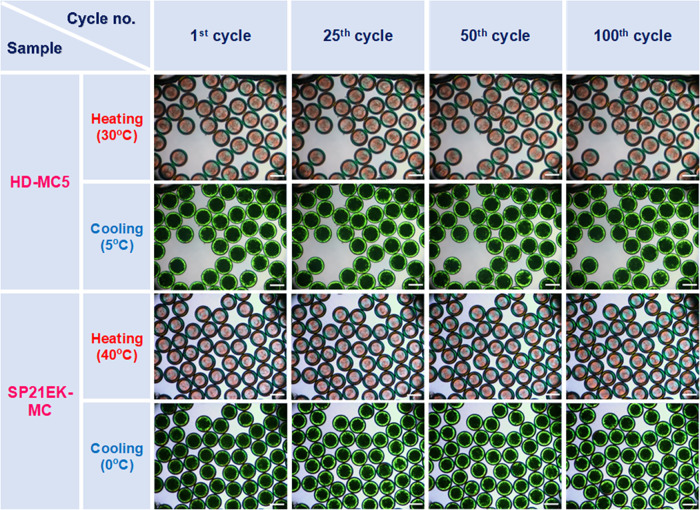
Polarization microscopy (thermo-optical) images showing the phase change in the core of capsules HD-MC4 and SP21EK-MC during 100 melting–crystallization cycles performed using a heating–cooling program. During melting, HD inside the microcapsule appears transparent and light red, while the shell shows a maltese cross structure in green and yellow, indicating the ordered structure of the polymerized NOA. After cooling, HD shows a dark green color, appearing black in the middle of the capsule where the layer thickness is highest. Scale bars show 250 μm.

Long-term exudation stability of PCM microcapsules during thermal cycling is one of the key requirements for their successful applications. The PCM inside microcapsules gets melted and then crystallizes again in each thermal cycle. Throughout the numerous cycles within the lifetime of the product, neither the melting/crystallization temperatures should change nor the encapsulated PCM should be released. Thermal cycling durability and the leakage performance of both HD and SP21EK microcapsules were studied using a thermo-optical polarizing microscope. For HD capsules, we selected the most fragile sample HD-MC5 with the thinnest shell that was most likely to leak. The two samples were placed on a temperature-controlled stage to undergo 100 repeating crystallization–melting cycles. A microscope camera with polarizing filters was used to monitor the crystallinity of the capsules in real time. Figure 9 displays the thermographic images of two microcapsule samples recorded after 1st, 25th, 50th, and 100th heating and cooling cycles. The narrow temperature range of 5–30 °C was applied to HD-based microcapsules as HD responds instantly to the temperature change and crystallizes quickly with a minor supercooling (as can be seen in the DSC thermogram in Figure 7a). On the other hand, a bit wider temperature range (0–40 °C) was given to the SP21EK-based microcapsules due to their inherent tendency of supercooling.

Movie S5 shows a single representative cycle of a sample HD-MC5 recorded during polarization thermographic analysis. Both samples show a rapid color change from red to green and green to red for heating and cooling stages, respectively. This phenomenon reflects a rapid thermal response of the encapsulated PCM. Interestingly, PCM melting (transition from green to red) occurred at nearly the same time for all of the capsules, while crystallization showed larger time variations between different capsules due to more stochastic nature of the nucleation process. Furthermore, the PCM core of all samples remained intact even after 100 cycles, proving that the NOA shell imparted excellent mechanical stability in spite of numerous volumetric changes of the core material during phase transitions. This confirms excellent thermal recyclability as PCM inside the microcapsules remained unchanged after 100 cycles. Moreover, both microcapsule samples retained their original shape and form without any morphological change across the whole test up to 100 cycles, confirming outstanding structural stability. These results firmly support the good sealing tightness of the NOA shell owing to its dense and nonporous structure as observed by SEM.

### 3.7 Photocatalytic Performance of Microcapsules

**Figure 10:**
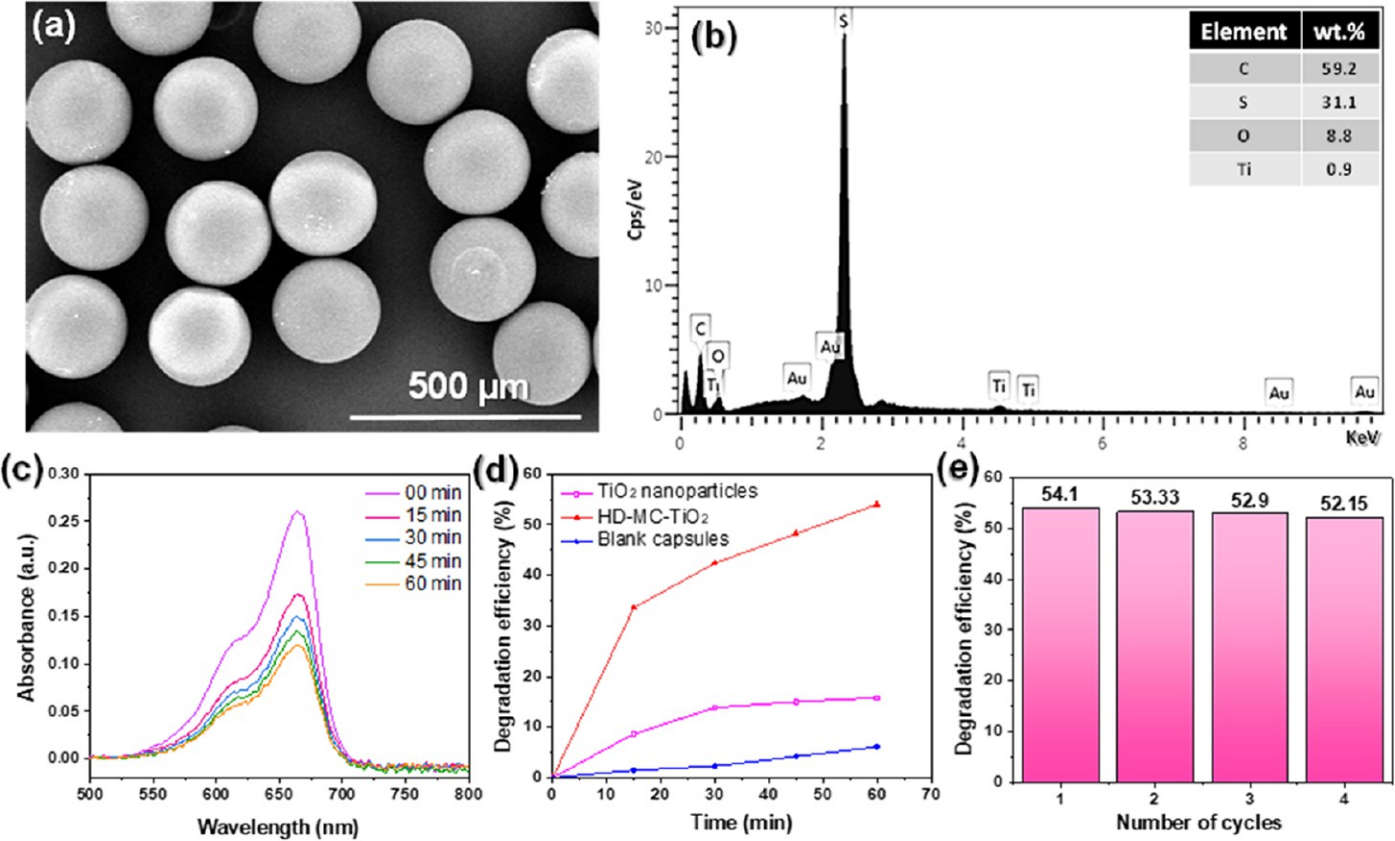
(a) SEM micrograph of TiO_2_-embedded HD microcapsules; (b) EDX spectrum of single microcapsule; (c) UV–visible absorbance spectra of the stirred MB solution mixed with HD-MC-TiO_2_ microcapsules; (d) MB degradation efficiency as a function of illumination time for samples of pure TiO_2_ NPs, HD-MC-TiO_2_, and blank microcapsules; and (e) recyclability test of the HD-MC-TiO_2_ sample. The initial concentration of MB was 1 ppm.

Application of PCM microcapsules can be significantly widened by imparting dual or multiple functionalities to polymeric shells. Multifunctionality can be attained in different ways: either the shell material itself is chosen for special properties or an existing shell material is enhanced by adding nanoparticles (NPs), or the morphology of the capsules is tailored to a specific shape.^42^ Crystalline titanium dioxide (TiO_2_) NPs have been widely researched as effective catalysts with high reactivity and excellent selectivity in pollution abatement applications. However, some drawbacks of TiO_2_ NPs such as a strong tendency to form aggregates, thereby minimizing their surface area, and the difficulty in separating them from the reaction system restrict their applicability.^43^ Immobilization of TiO_2_ NPs in a polymeric shell combines the advantages of catalyst recovery and reuse, high surface area, and mechanical and thermal stabilities. TiO_2_ NP-embedded microcapsules were prepared at the flow rates *Q*_i_ = 3.0 mL/h, *Q*_m_ = 1.5 mL/h, and *Q*_o_ = 20 mL/h (Movie S6). The middle phase contained 1 wt % TiO_2_ and was prepared by dispersing NPs in acetone first and then adding the suspension slowly to the NOA phase under sonication. The SEM image in Figure 10a revealed that the obtained microcapsules are highly monodisperse and compact. The capsules containing TiO_2_ NPs in the polymeric shell are opaque and white on optical microscopy image (Figure S7) due to high refractive index of TiO_2_ as compared to transparent, darker capsules with a pure NOA shell (Figure S5). Relatively uniform white opacity of the microcapsules in Figure S7 confirms that TiO_2_ is homogeneously distributed on the particle surfaces.

To quantify the TiO_2_ content, we examined the shell of a single microcapsule using EDX spectroscopy and determined that the content of the Ti element in the composite shell was 0.9 wt % (Figure 10b).

The photocatalytic degradation of methylene blue (MB) was examined as a model reaction for the potential application of the produced microcapsules. The microcapsules were tested against pristine TiO_2_ and blank capsules as reference systems. The pristine TiO_2_ NPs showed lower degradation activity (15.7% after 60 min) than TiO_2_-loaded capsules (>50% after 60 min). Very small degradation activity of blank microcapsules shows that MB cannot be degraded without TiO_2_ even in the presence of UV light and that the capsule material itself does not contribute to the degradation of MB (Figure 10d). The small fraction of MB removed by the blank capsules can be explained by physisorption of MB onto the shell material. This hypothesis was supported by the fact that no change in degradation activity was observed in the dark and under UV light for the blank microcapsules. On the other hand, TiO_2_-embedded capsules showed enhanced photocatalytic activity and caused a significant drop in the height of the MB absorbance peak from 0.26 a.u. to nearly 0.11 a.u. in 60 min (Figure 10c). Here, agglomeration of NPs was prevented by their immobilization within the host polymer, which reduced deactivation of activated TiO_2_ NPs by collision with ground-state TiO_2_ NPs.^19^ Under UV exposure, TiO_2_ excites its valence band electrons to the conduction band. This results in electron-deficient holes in the valence band and excess electrons in the conduction band. The electron-deficient holes in the valence band can produce OH^–^ radicals in water that subsequently oxidize MB.^44^

To investigate the reusability of TiO_2_-loaded microcapsules, the used microspheres were washed first with distilled water, then with acetone, and finally left to dry. The clean capsules were then resuspended in a fresh MB solution of the same concentration (1 ppm) and used in the next cycle. For each cycle, an adsorption spectrum was recorded and compared. As shown in Figure 10e, similar degradation rates of MB were found for each cycle: 54.1, 53.3, 52.9, and 52.1%. These results show that the produced capsules with embedded TiO_2_ can be effectively used as a photocatalyst and reused multiple times. The same approach can be used to load other catalysts into the NOA shell and produce various catalyst-loaded microencapsulated phase change materials, for example, for thermal control of exothermic reactions.^45^

### 3.8 Micromanipulation Analysis of Microcapsules

**Figure 11:**
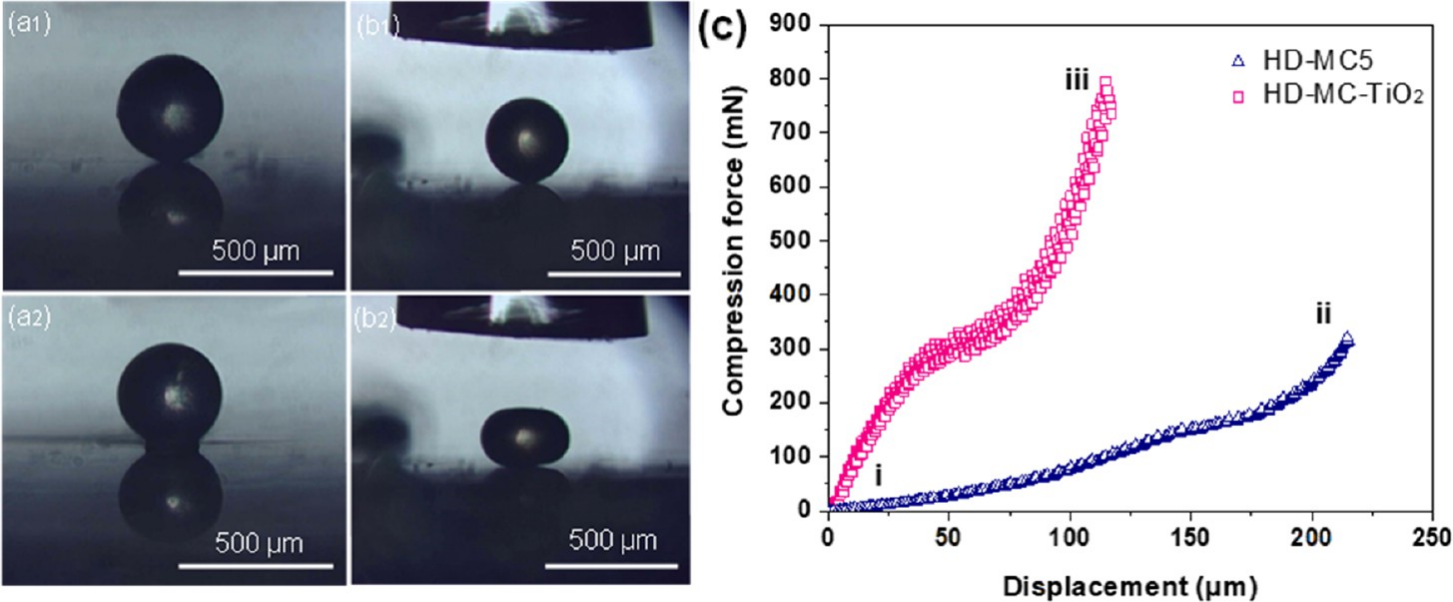
Microscopic images of a single microcapsule before and after compression *via* parallel plate compression: (a1, a2) HD-MC5; (b1, b2) HD-MC-TiO_2_; and (c) compression force vs displacement plot of samples HD-MC5 and HD-MC-TiO_2_.

Compression tests using a micromanipulation technique were carried out over 30 microcapsules of each sample to investigate the mechanical properties of the prepared PCM microcapsules. Figure 11a1–b2 displays the side-view images of microcapsule samples (HD-MC5 and HD-MC-TiO_2_) before and after compression and the results acquired from this analysis are summarized in Table 6.

**Table 6:** Mechanical Properties of HD-MC5 and HD-MC-TiO_2_ Samples Obtained from the Micromanipulation Analysis

sample	HD-MC5	HD-MC-TiO_2_
number of particles tested	30	30
shell thickness (μm)	19.5	20.1
rupture force (mN)	141.7 ± 12.8	NA
nominal rupture stress (MPa)	2.5 ± 0.2	NA
displacement at rupture (μm)	161.1 ± 7.0	NA
nominal rupture deformation (%)	60.0 ± 2.5	NA
Young’s modulus (MPa)	58.5 ± 1.4	224.4 ± 7.2
mean coefficient of determination, *R*^2^	0.89	0.92

For sample HD-MC5 (Figure 11a1,a2), all 30 microcapsules showed a clear rupture under compression with subsequent leakage of the HD core, which formed an oily meniscus between the contacting surface of the spherical microcapsule and the glass substrate. These microcapsules were found to exhibit a high shape recoverability once the compression probe had been released, although they looked smaller than their original size due to loss of their core content. In the case of HD-MC-TiO_2_, no microcapsule ruptured under compression, reflecting their much higher stiffness, but they showed permanent shell deformation after compression and release; see Figure 11b1,b2 and Movie S7.

Point (i) in the force vs displacement graph shown in Figure 11c represents the onset of compression for the TiO_2_-free microcapsule (HD-MC5), whereas segment (i)–(ii) describes the behavior of such a microcapsule at an increasingly high compressive force. This led to a progressive flattening of the microcapsule due to the compression force exerted onto the shell until a clear drop of the force signal was recorded at which HD was leaked out. The compression experiment was repeated 30 times, with randomly selected microcapsules for each repetition to generate statistically reliable results. Relatively narrow distributions of the rupture force, nominal rupture stress, displacement, and nominal strain at rupture, were obtained with an average value of 141.7 ± 12.8 mN, 2.5 ± 0.2 MPa, 161.1 ± 7.0 μm, and 60.1 ± 2.5%, respectively, in line with their narrow particle size distribution. Microcapsules produced *via* complex coacervation and *in situ* polymerization showed much broader distributions of particle sizes and mechanical properties.^18,45^ In the case of HD-MC-TiO_2_, a higher force has been generated by the probe compressing the capsule, yet no dramatic force drop was registered implying that no bursting point occurred.^46^ An inflection point of curve (iii) recorded at ∼50 μm of displacement suggests that the microcapsule has been transitioning from an elastic regime to a plastic region.

The compression force *versus* axial displacement curves of microcapsules is helpful to determine Young’s modulus. Young’s modulus is a crucial intrinsic material property parameter that describes the resistance (stiffness) of an elastic material undergoing elastic deformation under load. The apparent Young’s modulus (*E*_H_) of a solid microcapsule is described by the following equation^47^
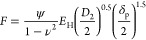
22where *F* is the compression force, ψ = 4/3 is the spherical shape coefficient, and ν is the Poisson ratio (ν = 0.5 for the incompressible polymer-based rubber-like matter). HD-MC-TiO_2_ and HD-MC5 were prepared at the same experimental conditions (Table 1) resulting in nearly the same shell thickness of 20.1 and 19.5 μm, respectively. However, HD-MC-TiO_2_ exhibited an elastic modulus of 224.4 ± 7.2 MPa (Table 6), whereas that of HD-MC5 was 58.5 ± 1.4 MPa, which is lower by about 74%. A significant increase in Young’s modulus of HD-MC-TiO_2_ is the result of incorporation of TiO_2_ NPs into the NOA shell. Pristine TiO_2_ possesses a very high Young’s modulus in the range of 110–150 GPa,^48^ which increases Young’s modulus of the composite NOA shell (*E*_c_) according to the equation: *E*_c_ = *E*_NOA_*v*_NOA_ + *E*_TiO_2__*v*_TiO_2__, where *v*_NOA_ and *v*_TiO_2__ are the volume fractions of NOA and TiO_2_ in the solid shell, while *E*_NOA_ and *E*_TiO_2__ are Young’s modulus of cured NOA and pristine TiO_2_, respectively.

## 4 Conclusions

A facile and reusable three-phase Lego-inspired glass capillary microfluidic device fabricated using CNC milling was successfully used for microencapsulation of phase change materials within a cured optical glue. This process proved to be highly energy-efficient, reproducible, and fast compared to the traditional microencapsulation processes. The Lego device could encapsulate hexadecane (organic PCM) and salt hydrate SP21EK (aqueous PCM) with 100% PCM yield. The maximum encapsulation efficiencies achieved for HD and SP21EK-based microcapsules were 65.4 and 44.3%, respectively. Highly monodisperse PCM microcapsules with controlled morphology, mechanical properties, and thermal energy storage capacity could be easily produced by adjusting phase flow rates in the microfluidic device and capillary geometry. Additionally, the morphology of the microcapsules and the encapsulation efficiency of PCMs could be accurately predicted using the derived correlations. Bifunctional microcapsules could be produced by incorporating TiO_2_ NPs in the shell of PCM microcapsules that exhibited concurrent photocatalysis and thermal energy storage properties.

Based on these results, several important properties of PCM microcapsules can be controlled, which can be employed for the proliferation of advanced energy storage materials. Importantly, the throughput of microcapsule production can be enhanced to several kilograms per hour by parallelizing multiple microchannels. It is envisaged that these PCM microcapsules can be useful as both heat sinks and heat sources for many micro/small-scale applications including thermoelectric generators, neuro-inspired computing devices, integrated circuits, and smart drug release systems. Moreover, bifunctional microcapsules can be applied in the biomedical field, residential buildings in polluted urban sites, industrial establishments, and smart textiles as thermal energy storage and depollution materials.

## Supporting Information Available

Engineering drawing of Lego blocks (Figure S1); dismantled microfluidic device with all fittings (Figure S2); microfluidic test rig setup for microencapsulation of PCMs using the Lego device (Figure S3); internal geometry of capillaries in the Lego device with all notations (Figure S4); optical microscopy images of HD-based microcapsules with transparent NOA shells (Figure S5); process of microfluidic encapsulation of salt hydrate SP21EK and SEM image of SP21EK-MC microcapsules (Figure S6); and optical microscopy image of TiO_2_ NP-embedded microcapsules with opaque NOA shells (Figure S7) (PDF)
Final PCM microcapsules obtained after drying (Movie S1) (MOV)
Core–shell droplets formation with controlled diameters using the Lego device (Movie S2) (MOV)
Core–shell droplets formation with controlled shell thicknesses using the Lego device (Movie S3) (MOV)
Process of microfluidic encapsulation of salt hydrate SP21EK (Movie S4) (MOV)
Polarization microscopy (thermo-optical) video showing a single phase change cycle of sample HD-MC4 (Movie S5) (MOV)
Microfluidic process for the synthesis of PCM microcapsules with the TiO_2_ NP-embedded shell (Movie S6) (MOV)
Analysis of mechanical properties by compressing a single HD-MC-TiO_2_ microcapsule via the micromanipulation technique (Movie S7) (MOV)

## Supplementary Material

am3c00281_si_001.mov

am3c00281_si_002.mov

am3c00281_si_003.mov

am3c00281_si_004.mov

am3c00281_si_005.mov

am3c00281_si_006.mov

am3c00281_si_007.mov

## Acronyms

HD: hexadecane
PCM: phase change material
NPs: nanoparticles
ID: inner diameter
OD: outer diameter
UV: ultraviolet
SH: salt hydrate
PVA: poly(vinyl alcohol)

## Nomenclature

*Ca*: capillary number
*F*: compression force (N)
*D*_2_: outer diameter of a microcapsule, m
*d*_i_: inner diameter of a droplet/particle, m
*d*_o_: outer diameter of a droplet, m
*D*_ii_: inner diameter of the injection capillary orifice, m
*D*_io_: outer diameter of the inner capillary, m
*D*_ci_: inner diameter of the collection capillary orifice, m
*D*_bi_: inner diameter of a big (outer) capillary, m
*D*_j_: diameter of two-phase liquid jet in microfluidic device, m
Δ*H*: melting enthalpy, J/kg
*Q*_i_: flow rate of the inner phase, m^3^/s
*Q*_m_: flow rate of the middle phase, m^3^/s
*Q*_o_: flow rate of the outer phase, m^3^/s

## Greek Symbols

δ: shell thickness, m
*P*: density, kg/m^3^
μ: viscosity, Pa·s
σ: interfacial tension, N/m
ζ: empirical parameter
θ: contact angle, rad
ψ: spherical shape coefficient
ν: Poisson ratio

## Subscripts

a: acetone
NOA: NOA 81
d: droplet
p: particle

## References

[ref1] Carrillo A. J.; González-Aguilar J.; Romero M.; Coronado J. M. Solar Energy on Demand: A Review on High Temperature Thermochemical Heat Storage Systems and Materials. Chem. Rev. 2019, 119, 4777–4816. 10.1021/acs.chemrev.8b00315. 30869873

[ref2] Safari A.; Saidur R.; Sulaiman F. A.; Xu Y.; Dong J. A Review on Supercooling of Phase Change Materials in Thermal Energy Storage Systems. Renewable Sustainable Energy Rev. 2017, 70, 905–919. 10.1016/j.rser.2016.11.272.

[ref3] Peng H.; Zhang D.; Ling X.; Li Y.; Wang Y.; Yu Q.; She X.; Li Y.; Ding Y. n-Alkanes Phase Change Materials and Their Microencapsulation for Thermal Energy Storage: A Critical Review. Energy Fuels 2018, 32, 7262–7293. 10.1021/acs.energyfuels.8b01347.

[ref4] Parvate S.; Chattopadhyay S. Complex Polymeric Microstructures with Programmable Architecture via Pickering Emulsion-Templated In-Situ Polymerization. Langmuir 2022, 38, 1406–1421. 10.1021/acs.langmuir.1c02572. 35051332

[ref5] Parvate S.; Mahanwar P. Insights into the Preparation of Water-Based Acrylic Interior Decorative Paint: Tuning Binder’s Properties by Self-Crosslinking of Allyl Acetoacetate - Hexamethylenediamine. Prog. Org. Coat. 2019, 126, 142–149. 10.1016/j.porgcoat.2018.10.014.

[ref6] Parvate S.; Singh J.; Reddy Vennapusa J.; Dixit P.; Chattopadhyay S. Copper Nanoparticles Interlocked Phase-Change Microcapsules for Thermal Buffering in Packaging Application. J. Ind. Eng. Chem. 2021, 102, 69–85. 10.1016/j.jiec.2021.06.029.

[ref7] Liu H.; Wang X.; Wu D. Fabrication of Graphene/TiO_2_/Paraffin Composite Phase Change Materials for Enhancement of Solar Energy Efficiency in Photocatalysis and Latent Heat Storage. ACS Sustainable Chem. Eng. 2017, 5, 4906–4915. 10.1021/acssuschemeng.7b00321.

[ref8] Huang Y. T.; Zhang H.; Wan X. J.; Chen D. Z.; Chen X. F.; Ye X.; Ouyang X.; Qin S. Y.; Wen H. X.; Tang J. N. Carbon Nanotube-Enhanced Double-Walled Phase-Change Microcapsules for Thermal Energy Storage. J. Mater. Chem. A 2017, 5, 7482–7493. 10.1039/C6TA09712J.

[ref9] Mu X. T.; Li Y.; Ju X. J.; Yang X. L.; Xie R.; Wang W.; Liu Z.; Chu L. Y. Microfluidic Fabrication of Structure-Controlled Chitosan Microcapsules via Interfacial Cross-Linking of Droplet Templates. ACS Appl. Mater. Interfaces 2020, 12, 57514–57525. 10.1021/acsami.0c14656. 33301686

[ref10] Utada A. S.; Lorenceau E.; Link D. R.; Kaplan P. D.; Stone H. A.; Weitz D. A. Monodisperse Double Emulsions Generated from a Microcapillary Device. Science 2005, 308, 537–541. 10.1126/science.1109164. 15845850

[ref11] Takeuchi S.; Garstecki P.; Weibel D. B.; Whitesides G. M. An Axisymmetric Flow-Focusing Microfluidic Device. Adv. Mater. 2005, 17, 1067–1072. 10.1002/adma.200401738.

[ref12] Lone S.; Lee H. M.; Kim G. M.; Koh W. G.; Cheong I. W. Facile and Highly Efficient Microencapsulation of a Phase Change Material Using Tubular Microfluidics. Colloids Surf., A 2013, 422, 61–67. 10.1016/j.colsurfa.2013.01.035.

[ref13] Fu Z.; Su L.; Li J.; Yang R.; Zhang Z.; Liu M.; Li J.; Li B. Elastic Silicone Encapsulation of n-Hexadecyl Bromide by Microfluidic Approach as Novel Microencapsulated Phase Change Materials. Thermochim. Acta 2014, 590, 24–29. 10.1016/j.tca.2014.06.008.

[ref14] Han X.; Kong T.; Zhu P.; Wang L. Microfluidic Encapsulation of Phase-Change Materials for High Thermal Performance. Langmuir 2020, 36, 8165–8173. 10.1021/acs.langmuir.0c01171. 32575990

[ref15] Hao G.; Yu C.; Chen Y.; Liu X.; Chen Y. Controlled Microfluidic Encapsulation of Phase Change Material for Thermo-Regulation. Int. J. Heat Mass Transfer 2022, 190, 122738 10.1016/j.ijheatmasstransfer.2022.122738.

[ref16] Ryu S. A.; Hwang Y. H.; Oh H.; Jeon K.; Lee J. H.; Yoon J.; Lee J. B.; Lee H. Biocompatible Wax-Based Microcapsules with Hermetic Sealing for Thermally Triggered Release of Actives. ACS Appl. Mater. Interfaces 2021, 13, 36380–36387. 10.1021/acsami.1c04652. 34255487

[ref17] Wang J.; Hahn S.; Amstad E.; Vogel N. Tailored Double Emulsions Made Simple. Adv. Mater. 2022, 34, 2107338 10.1002/adma.202107338. 34706112

[ref18] Zhang Y.; Baiocco D.; Mustapha A. N.; Zhang X.; Yu Q.; Wellio G.; Zhang Z.; Li Y. Hydrocolloids: Nova Materials Assisting Encapsulation of Volatile Phase Change Materials for Cryogenic Energy Transport and Storage. Chem. Eng. J. 2020, 382, 123028 10.1016/j.cej.2019.123028.

[ref19] Parvate S.; Singh J.; Dixit P.; Vennapusa J. R.; Maiti T. K.; Chattopadhyay S. Titanium Dioxide Nanoparticle-Decorated Polymer Microcapsules Enclosing Phase Change Material for Thermal Energy Storage and Photocatalysis. ACS Appl. Polym. Mater. 2021, 3, 1866–1879. 10.1021/acsapm.0c01410.

[ref20] Wang W.; Li B. Y.; Zhang M. J.; Su Y. Y.; Pan D. W.; Liu Z.; Ju X. J.; Xie R.; Faraj Y.; Chu L. Y. Microfluidic Emulsification Techniques for Controllable Emulsion Production and Functional Microparticle Synthesis. Chem. Eng. J. 2023, 452, 139277 10.1016/j.cej.2022.139277.

[ref21] Vilanova N.; Rodríguez-Abreu C.; Fernández-Nieves A.; Solans C. Fabrication of Novel Silicone Capsules with Tunable Mechanical Properties by Microfluidic Techniques. ACS Appl. Mater. Interfaces 2013, 5, 5247–5252. 10.1021/am4010896. 23659612

[ref22] Giteru S. G.; Azam Ali M.; Oey I. Elucidating the pH Influence on Pulsed Electric Fields-Induced Self-Assembly of Chitosan-Zein-Poly(Vinyl Alcohol)-Polyethylene Glycol Nanostructured Composites. J. Colloid Interface Sci. 2021, 588, 531–546. 10.1016/j.jcis.2020.12.075. 33429349

[ref23] Leister N.; Vladisavljević G. T.; Karbstein H. P. Novel Glass Capillary Microfluidic Devices for the Flexible and Simple Production of Multi-Cored Double Emulsions. J. Colloid Interface Sci. 2022, 611, 451–461. 10.1016/j.jcis.2021.12.094. 34968964

[ref24] Bandulasena M. V.; Vladisavljević G. T.; Benyahia B. Versatile Reconfigurable Glass Capillary Microfluidic Devices with Lego-Inspired Blocks for Drop Generation and Micromixing. J. Colloid Interface Sci. 2019, 542, 23–32. 10.1016/j.jcis.2019.01.119. 30721833

[ref25] Norland Optical Adhesive 81, www.norlandproducts.com (accessed on 2nd May 2022).

[ref26] Chiou B.-S.; English R. J.; Khan S. A. Rheology and Photo-Cross-Linking of Thiol-Ene Polymers. Macromolecules 1996, 29, 5368–5374. 10.1021/ma960383e.

[ref27] Nabavi S. A.; Vladisavljević G. T.; Bandulasena M. V.; Arjmandi-Tash O.; Manović V. Prediction and Control of Drop Formation Modes in Microfluidic Generation of Double Emulsions by Single-Step Emulsification. J. Colloid Interface Sci. 2017, 505, 315–324. 10.1016/j.jcis.2017.05.115. 28601740

[ref28] Omotika S. T. On the Instability of a Cylindrical Thread of a Viscous Liquid Surrounded by Another Viscous Fluid. Proc. R. Soc. A 1935, 150, 322–337. 10.1098/rspa.1935.0104.

[ref29] Ekanem E. E.; Nabavi S. A.; Vladisavljević G. T.; Gu S. Structured Biodegradable Polymeric Microparticles for Drug Delivery Produced Using Flow Focusing Glass Microfluidic Devices. ACS Appl. Mater. Interfaces 2015, 7, 23132–23143. 10.1021/acsami.5b06943. 26423218

[ref30] Wu Z.; Werner J. G.; Weitz D. A. Microfluidic Fabrication of Phase-Inverted Microcapsules with Asymmetric Shell Membranes with Graded Porosity. ACS Macro Lett. 2021, 10, 116–121. 10.1021/acsmacrolett.0c00858. 35548985

[ref31] Tu F.; Lee D. Controlling the Stability and Size of Double-Emulsion-Templated Poly(Lactic- *co* -Glycolic) Acid Microcapsules. Langmuir 2012, 28, 9944–9952. 10.1021/la301498f. 22667691

[ref32] Dixit P.; Reddy V. J.; Parvate S.; Balwani A.; Singh J.; Maiti T. K.; Dasari A.; Chattopadhyay S. Salt Hydrate Phase Change Materials: Current State of Art and the Road Ahead. J. Energy Storage 2022, 51, 104360 10.1016/j.est.2022.104360.

[ref33] Kenisarin M. M. High-Temperature Phase Change Materials for Thermal Energy Storage. Renewable Sustainable Energy Rev. 2010, 14, 955–970. 10.1016/j.rser.2009.11.011.

[ref34] Deng Y.; Li J.; Deng Y.; Nian H.; Jiang H. Supercooling Suppression and Thermal Conductivity Enhancement of Na_2_HPO_4_·12H_2_O/Expanded Vermiculite Form-Stable Composite Phase Change Materials with Alumina for Heat Storage. ACS Sustainable Chem. Eng. 2018, 6, 6792–6801. 10.1021/acssuschemeng.8b00631.

[ref35] Sarı A.; Alkan C.; Altintaş A. Preparation, Characterization and Latent Heat Thermal Energy Storage Properties of Micro-Nanoencapsulated Fatty Acids by Polystyrene Shell. Appl. Therm. Eng. 2014, 73, 1160–1168. 10.1016/j.applthermaleng.2014.09.005.

[ref36] Zhan S.; Chen S.; Chen L.; Hou W. Preparation and Characterization of Polyurea Microencapsulated Phase Change Material by Interfacial Polycondensation Method. Powder Technol. 2016, 292, 217–222. 10.1016/j.powtec.2016.02.007.

[ref37] Li J.; Liu H.; Wang X.; Wu D. Development of Thermoregulatory Enzyme Carriers Based on Microencapsulated *n*-Docosane Phase Change Material for Biocatalytic Enhancement of Amylases. ACS Sustainable Chem. Eng. 2017, 5, 8396–8406. 10.1021/acssuschemeng.7b02200.

[ref38] Konuklu Y.; Paksoy H. O.; Unal M.; Konuklu S. Microencapsulation of a Fatty Acid with Poly(Melamine-Urea-Formaldehyde). Energy Convers. Manage. 2014, 80, 382–390. 10.1016/j.enconman.2014.01.042.

[ref39] Fang Y.; Zou T.; Liang X.; Wang S.; Liu X.; Gao X.; Zhang Z. Self-Assembly Synthesis and Properties of Microencapsulated n-Tetradecane Phase Change Materials with a Calcium Carbonate Shell for Cold Energy Storage. ACS Sustainable Chem. Eng. 2017, 5, 3074–3080. 10.1021/acssuschemeng.6b02758.

[ref40] Salaün F.; Devaux E.; Bourbigot S.; Rumeau P. Influence of the Solvent on the Microencapsulation of an Hydrated Salt. Carbohydr. Polym. 2010, 79, 964–974. 10.1016/j.carbpol.2009.10.027.

[ref41] Fu W.; Zou T.; Liang X.; Wang S.; Gao X.; Zhang Z.; Fang Y. Characterization and Thermal Performance of Microencapsulated Sodium Thiosulfate Pentahydrate as Phase Change Material for Thermal Energy Storage. Solar Energy Mater. Solar Cells 2019, 193, 149–156. 10.1016/j.solmat.2019.01.007.

[ref42] Chen X.; Tang Z.; Liu P.; Gao H.; Chang Y.; Wang G. Smart Utilization of Multifunctional Metal Oxides in Phase Change Materials. Matter 2020, 3, 708–741. 10.1016/j.matt.2020.05.016.

[ref43] Yamada Y.; Mizutani M.; Nakamura T.; Yano K. Mesoporous Microcapsules with Decorated Inner Surface: Fabrication and Photocatalytic Activity. Chem. Mater. 2010, 22, 1695–1703. 10.1021/cm9031072.

[ref44] Parvate S.; Dixit P.; Chattopadhyay S. Hierarchical Polymeric Hollow Microspheres with Size Tunable Single Holes and Their Application as Catalytic Microreactor. Colloid Polym. Sci. 2022, 300, 1101–1109. 10.1007/s00396-022-05008-7.

[ref45] Zhang Y.; Mustapha A. N.; Zhang X.; Baiocco D.; Wellio G.; Davies T.; Zhang Z.; Li Y. Improved Volatile Cargo Retention and Mechanical Properties of Capsules via Sediment-Free in Situ Polymerization with Cross-Linked Poly(Vinyl Alcohol) as an Emulsifier. J. Colloid Interface Sci. 2020, 568, 155–164. 10.1016/j.jcis.2020.01.115. 32088446

[ref46] Pan X.; Mercadé-Prieto R.; York D.; Preece J. A.; Zhang Z. Structure and Mechanical Properties of Consumer-Friendly PMMA Microcapsules. Ind. Eng. Chem. Res. 2013, 52, 11253–11265. 10.1021/ie303451s.

[ref47] Dintwa E.; Tijskens E.; Ramon H. On the Accuracy of the Hertz Model to Describe the Normal Contact of Soft Elastic Spheres. Granular Matter 2008, 10, 209–221. 10.1007/s10035-007-0078-7.

[ref48] Borgese L.; Gelfi M.; Bontempi E.; Goudeau P.; Geandier G.; Thiaudière D.; Depero L. E. Young Modulus and Poisson Ratio Measurements of TiO_2_ Thin Films Deposited with Atomic Layer Deposition. Surf. Coat. Technol. 2012, 206, 2459–2463. 10.1016/j.surfcoat.2011.10.050.

