# Supplementary material for: Lego-Inspired Glass Capillary Microfluidic Device: A Technique for Bespoke Microencapsulation of Phase Change Materials
Source: ACS Appl Mater Interfaces. 2023 Mar 24;15(13):17195–210. doi: 10.1021/acsami.3c00281 (PMC10080541; doi:10.1021/acsami.3c00281)
Supplement: Supplementary file 8 — am3c00281_si_008.pdf [file am3c00281_si_008.pdf]

Supporting Information

Lego-Inspired Glass Capillary Microfluidic Device: A Technique for Bespoke Microencapsulation of Phase Change Materials

Sumit Parvate [a,b], Goran T. Vladisavljevic [a]*, Nico Leister [c], Alexandros Spyrou [a],

Guido Bolognesi [a], Daniele Baiocco [d], Zhibing Zhang [d], Sujay Chattopadhyay [b]

[a] Department of Chemical Engineering, Loughborough University, Loughborough LE11 3TU, United Kingdom
[b] Polymer and Process Engineering, Indian Institute of Technology, Roorkee, Saharanpur 247001, India
[c] Institute of Process Engineering in Life Sciences, Karlsruhe Institute of Technology, 76131, Karlsruhe, Germany
[d] School of Chemical Engineering, University of Birmingham, Birmingham B15 2TT, United Kingdom

Correspondence to: Dr. Goran Vladisavljević (Email ID: G.Vladisavljevic@lboro.ac.uk)

Supplemental material S1

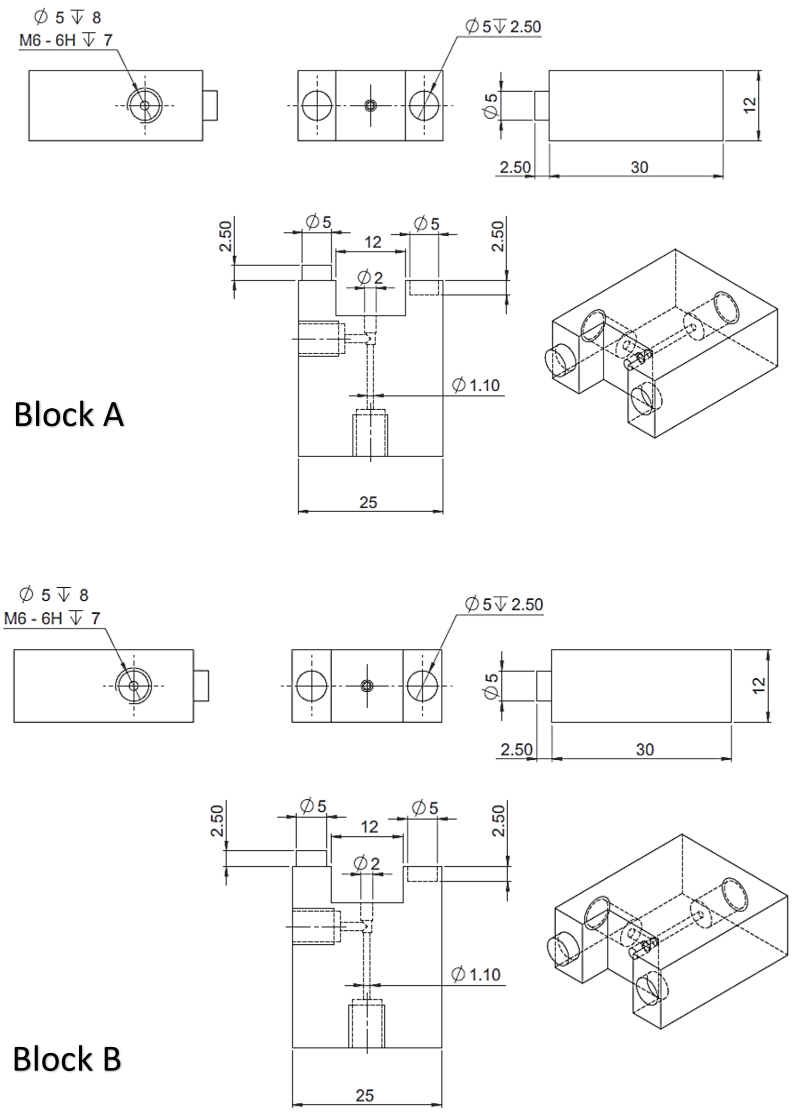

Figure S1. Engineering drawing of Lego blocks.

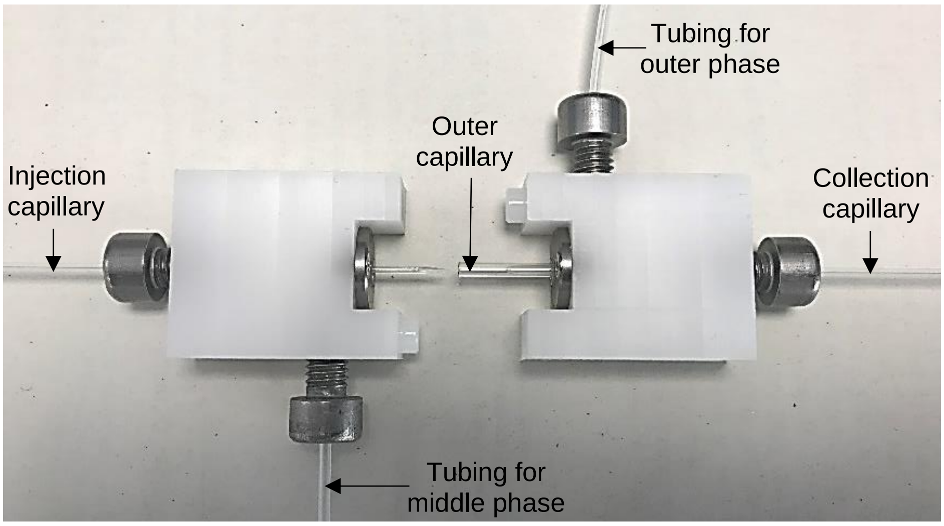

Figure S2. A dismantled microfluidic device with interchangeable Lego-inspired blocks. Glass capillaries, polyethylene tubing, and stainless-steel tube connectors are also shown.

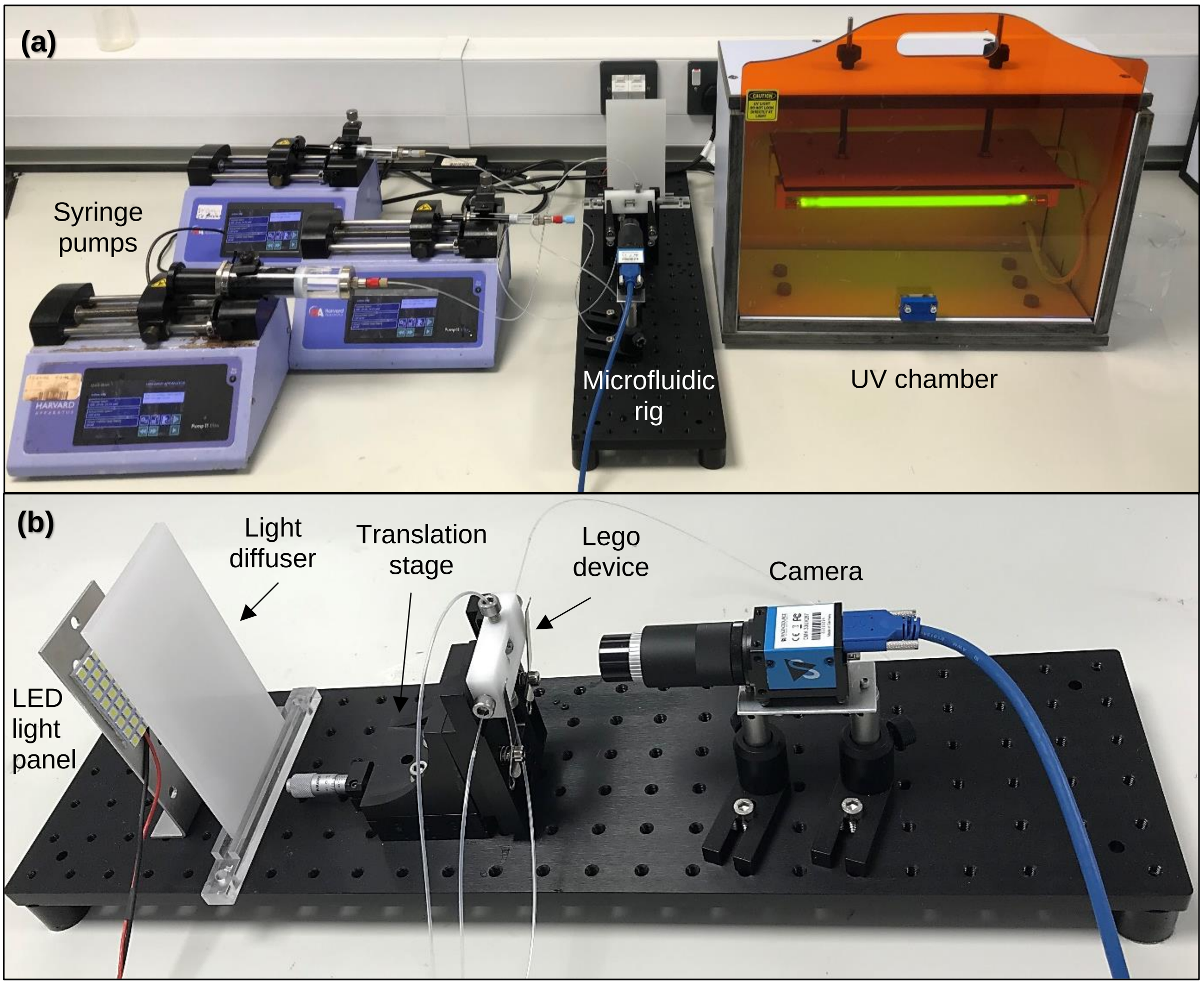

Figure S3. (a) Complete experimental set-up for microencapsulation of PCMs using Lego-inspired microfluidic device; (b) Side view of microfluidic test rig.

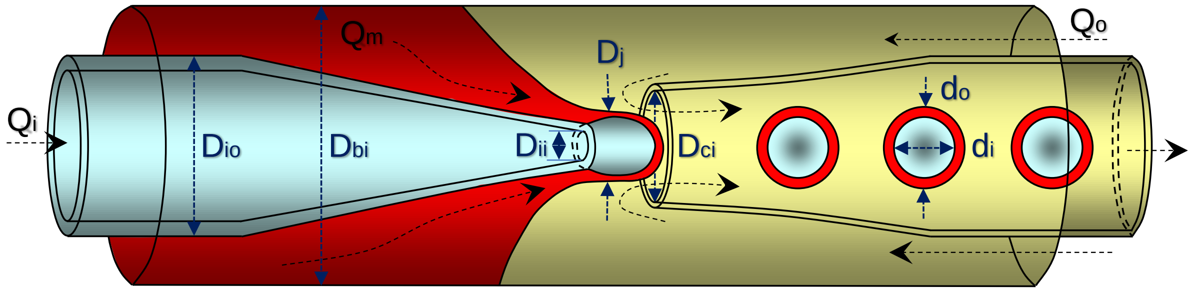

Figure S4. Scheme showing the internal geometry of capillaries in the Lego microfluidic device, fluid streams and generated core-shell droplets with all notations.

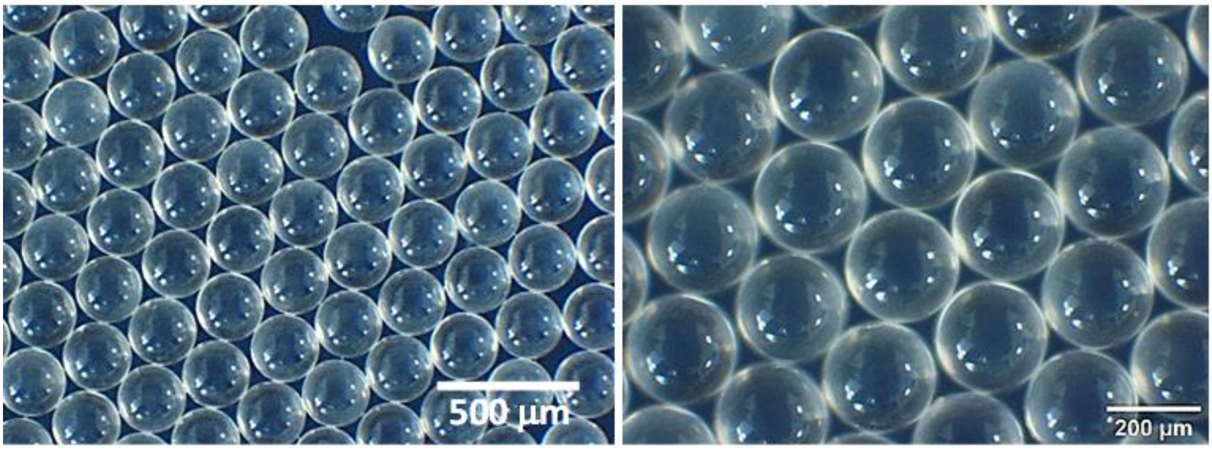

Figure S5. Optical microscopy images of HD-based microcapsules with transparent NOA shells at different magnifications.

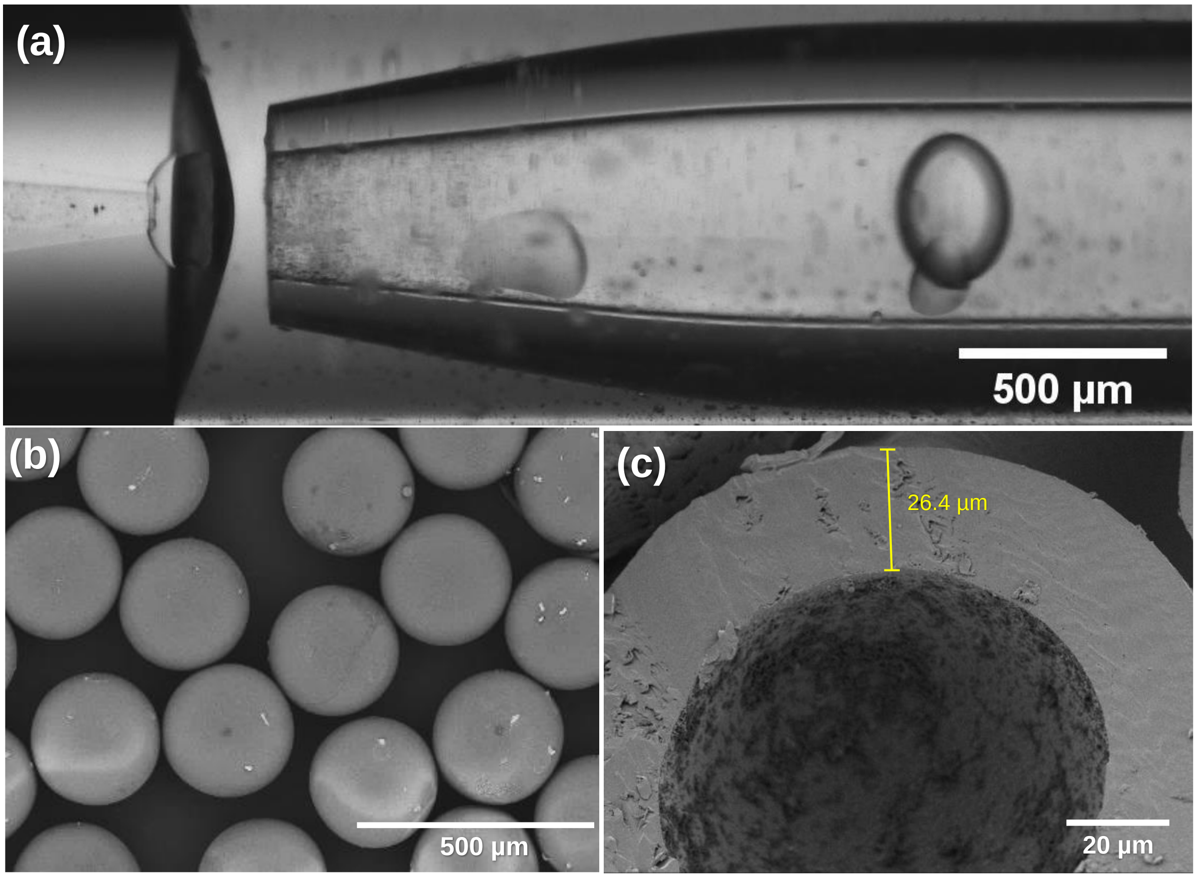

Figure S6. (a) Microfluidic process for the synthesis of microcapsules enclosing salt hydrate SP21EK (SP21EK-MC); (b) SEM image of SP21EK-MC microcapsules; and (c) SEM image of cross-sectioned SP21EK-MC microcapsule.

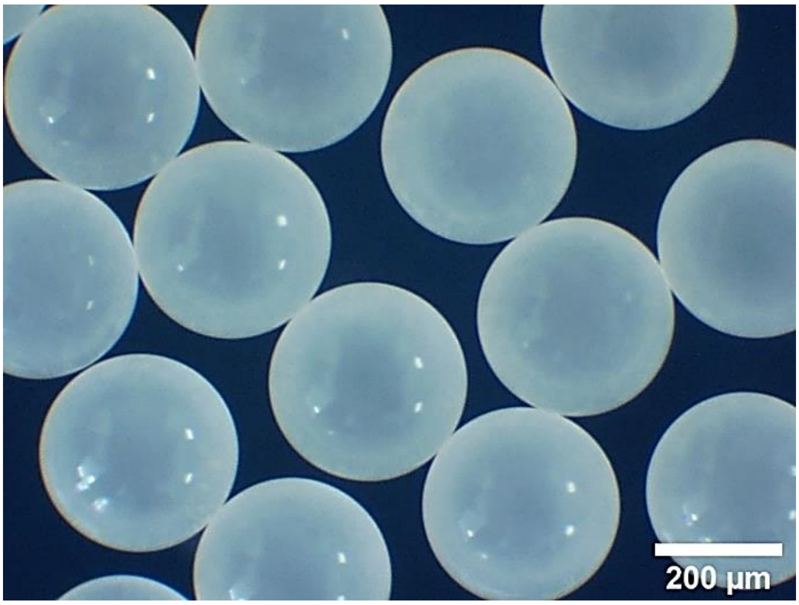

Figure S7. Optical microscopy image of HD-based microcapsules with opaque NOA shells confirming the presence of embedded TiO_2 NPs. Relatively uniform opacity confirms that TiO_2 is homogeneously distributed on the particle surfaces.

Supplemental material S2

Movie S1. The free-flowing final PCM microcapsules obtained after drying.

Movie S2. Double emulsions (O/O/W) with controlled droplet diameters formed using the three-phase Lego microfluidic device at Q_i = 1.5 mL/h, Q_m = 1.5 mL/h, Q_o = 20 mL/h using orifice diameters $D_{ii} = 50$ µm and $D_{ci} = 200$ µm (upper movie, HD-MC1); $D_{ii} = 100$ µm and $D_{ci} = 400$ µm (middle movie, HD-MC2); and $D_{ii} = 200$ µm and $D_{ci} = 500$ µm (lower movie, HD-MC3). The videos were slowed down 40 times.

Movie S3. Double emulsions (O/O/W) with controlled shell thicknesses formed using the three-phase Lego microfluidic device at orifice diameters $D_{ii} = 100$ µm and $D_{ci} = 400$ µm and flow rates Q_i = 0.75 mL/h, $Q_m =$ 1.5 mL/h, $Q_o =$ 20 mL/h (upper movie, HD-MC4); $Q_i =$ 1.5 mL/h, $Q_m =$ 1.5 mL/h, $Q_o =$ 20 mL/h (middle movie, HD-MC2); and $Q_i =$ 3 mL/h, $Q_m =$ 1.5 mL/h, $Q_o =$ 20 mL/h (lower movie, HD-MC5). The videos were slowed down 40 times.

Movie S4. Microfluidic process for synthesis of microcapsules enclosing salt hydrate SP21EK (SP21EK-MC) at $D_{ii} = 100$ µm and $D_{ci} = 400$ µm and flow rates $Q_i =$ 1.5 mL/h, $Q_m =$ 1.5 mL/h, $Q_o =$ 20 mL/h. The video was slowed down 40 times.

Movie S5. Polarization microscopy (thermo-optical) video showing a single representative cycle of phase change of sample HD-MC4. During melting, HD inside the microcapsule appears transparent in the pictures while the shell shows a maltese cross structure in green and yellow, indicating the ordered structure of the polymerized NOA. During cooling, HD shows a dark green colour, appearing black in the middle of the capsule where the layer thickness is highest.

Movie S6. Real-time microfluidic process for synthesis of TiO_2-HD-MC (PCM microcapsules with TiO_2 NPs embedded shell) at $D_{ii} = 100$ µm and $D_{ci} = 400$ µm and flow rates $Q_i =$ 3 mL/h, $Q_m =$ 1.5 mL/h, and $Q_o =$ 20 mL/h.

Movie S7. Analysis of mechanical properties by compressing a single HD-MC-TiO_2 microcapsule *via* micromanipulation technique.
